# Gut REG3γ-Associated *Lactobacillus* Induces Anti-inflammatory Macrophages to Maintain Adipose Tissue Homeostasis

**DOI:** 10.3389/fimmu.2017.01063

**Published:** 2017-09-04

**Authors:** Yugang Huang, HouBao Qi, Zhiqian Zhang, Enlin Wang, Huan Yun, Hui Yan, Xiaomin Su, Yingquan Liu, Zenzen Tang, Yunhuan Gao, Wencong Shang, Jiang Zhou, Tianze Wang, Yongzhe Che, Yuan Zhang, Rongcun Yang

**Affiliations:** ^1^State Key Laboratory of Medicinal Chemical Biology, Nankai University, Tianjin, China; ^2^Key Laboratory of Bioactive Materials Ministry of Education, Nankai University, Tianjin, China; ^3^Department of Immunology, Nankai University School of Medicine, Nankai University, Tianjin, China

**Keywords:** REG3γ, macrophage, STAT3, *Lactobacillus*, gut microbiota

## Abstract

Gut microbiota may not only affect composition of local immune cells but also affect systemic immune cells. However, it is not completely clear how gut microbiota modulate these immune systems. Here, we found that there exist expanded macrophage pools in *huREG3*γ^*tgIEC*^ mice. REG3γ-associated *Lactobacillus*, which is homology to *Lactobacillus Taiwanese*, could enlarge macrophage pools not only in the small intestinal lamina propria but also in the spleen and adipose tissues. STAT3-mediated signal(s) was a critical factor in the *Lactobacillus*-mediated anti-inflammatory macrophages. We also offered evidence for critical cellular links among REG3γ-associated *Lactobacillus*, tissue macrophages, and obesity diseases. Anti-inflammatory macrophages in the lamina propria, which are induced by REG3γ-associated *Lactobacillus*, may migrate into adipose tissues and are involved in resistance against high-fat diet-mediated obesity. Thus, REG3γ-associated *Lactobacillus*-induced anti-inflammatory macrophages in gut tissues may play a role in adipose tissue homeostasis.

## Introduction

The alteration of gut homeostasis is often associated with the onset of metabolic diseases such as obesity (1–4). The gut immune system plays an essential role in the maintenance of gut immune homeostasis with resident microbial communities (5, 6). The gut-resident macrophages, one of the largest populations of macrophages (Mϕ), are a main contributor of gut homeostasis (7–9). Macrophages may harbor a variety of surface and intracellular receptors, multiple signal transduction pathways, and complex, adaptable arrays of gene expression (10). There mainly are two main macrophage groups designated as M1 (inflammatory Mϕ) and M2 (anti-inflammatory Mϕ). Gut monocyte-derived macrophages are sessile cells and anti-inflammatory in nature (11). These gut tissue-resident macrophages are generally characterized as MHCII(+) F4/80(+)CD11c(+) CD11b(+)CD103(−)CX3CRl(+)CD64(+) cells (6). They may produce an anti-inflammatory cytokine interleukin (IL)-10 and induce regulatory T cells (8). These resident Mϕs maintain their anti-inflammatory signature even when stimulated *via* Toll-like receptors (TLRs) and other triggers (12). However, it is unclear how gut maintains gut macrophages in anti-inflammatory situation and/or how to cause accumulation of these macrophages in gut tissues.

The mounting evidence indicates importance of gut microbiota/immune cell interactions in immune homeostasis. Intestinal microbiota and/or their metabolitics may actively regulate the host’s immune system. The interactions with different components of microbiota are crucial to the establishment and development of gut immune cell populations in gut tissues (5, 13–16). Thus, it is possible that the gut anti-inflammatory macrophages in gut tissues may be induced by gut microbiota and/or their metabolitics. Intestine-resident macrophages are derived from monocytes, and the monocytes may differentiate into tissue macrophages in the healthy intestinal lamina propria (LP) (9, 17). Recent studies have showed that microbiota may regulate the gut macrophage pools (9). Administration of broad-spectrum antibiotics to conventionally reared wild-type (WT) mice could result in small, but significant reductions in the numbers of the Ly6C(hi)MHCII(−) and Ly6C(+)MHCII(+) monocyte-like subsets in the colon (9). All the monocyte-macrophage subsets were showed to be strikingly fewer in the colon of adult germ-free (GF) mice compared with conventionally housed controls (9). These studies indicate that gut microbiota may be necessary to maintain the homeostasis of intestinal-resident anti-inflammatory macrophages.

REG3γ is a secreted antibacterial lectin, one class of antimicrobial peptides that are expressed in the gut epithelial Paneth cells. Mouse Reg3γ and its human counterpart are direct antimicrobial proteins that bind to their bacterial targets *via* interactions with peptidoglycan carbohydrate (18). Furthermore, knockout of REG3γ affects mucus distribution and promotes mucosal inflammatory responses to the microbiota and enteric pathogens in the ileum (19). Here, we found that gut anti-inflammatory macrophage pools in gut REG3γ-overexpressing mice are significantly larger than WT mice. By using human REG3γ transgenic (*huREG3*γ*^tgIEC^* mice) mice, we identify one strain of bacteria, named as *Lactobacillus NK318.1*, which may directly modulate the differentiation of gut macrophages and enlarge gut macrophage pools not only in gut tissues but also in spleen and adipose tissues. Meanwhile, we also found a critical cellular link among REG3γ-associated *Lactobacillus*, tissue macrophages, and obesity diseases. Gut REG3γ-associated *Lactobacillus* may have capacities to impede the onset of obesity through increased gut anti-inflammatory macrophages.

## Materials and Methods

### Reagents

Anti-mouse phosphor-STAT3 (EP2147Y, Abcam), phosphor-NF-κB (pp65) (sc-52401, Santa), phosphor-JNK (pJNK)(9H8), phosphor-ERK (pERK)(197G2), STAT3 (124H6, Cell Signaling), NF-κBp65 (sc8008, Santa), JNK (SC), ERK(137F5), and β-actin (sc-47778, Santa) were purchased. FITC-, PE-, APC- APC/cy7-, PerCP/Cy5.5-, or PE/cy7-conjugated anti-mouse CD4 (RM4-5, Biolegend), CD8 (ZUT270.5, Biolegend), CD45.1 (30-F11, Biolegend), MHCII (I-A/I-E, M5/114.115.2, Biolegend), CD11c (MCA1441GA, Biolegend), CD11b (M1/70, eBioscience), F4/80 (BM8, Biolegend), CD206 (CO68C2), Gr-1(RB6-8C5, eBioscience), IL-10 (JES5-16E3, eBioscience), Foxp3 (MF23, eBioscience), IL-22 (140301, R&D system), IFNγ (XMG1.2, Biolegend), RORγt (AFKJS-9, eBioscience), IL-17A (eBio17B7, eBioscience), and CD3ζ (H146-968, Abcam) antibodies were purchased. Anti- REG3γ (PA517, Thermo) and anti-MUC2 (H300, Santa) were also purchased. FITC-, PE-, APC- APC/cy7-, PerCP/Cy5.5-, or PE/cy7-conjugated isotypic controls were from Biolegend. RPMI1640, DMEM, *fetal bovine serum* (FBS), and antibiotics were obtained from HyClone. Alexa fluor 488-conjugated goat anti-rabbit IgGH&L and Alexa fluor 594-conjugated goat anti-rabbit IgGH&L (Abcam) were also purchased. 7-AAD was from Abcam.

*Lactobacillus reuteri* (ATCC PTA 4659) was a gift of BioGaaia, Sweden.

### Mice, HuREG3γ Transgenic Mice

Four- to six-week-old male or female C57BL/6, OT-I, OT-II, CD45.1, and *TLR2^−/−^* mice were obtained from Nanjing Animal Center. The FVB-GFP/Fluc mice were kindly offered by Prof. Zongjin Li in Nankai University. C57BL/6 GF mice were generated by Shanghai SLAC Laboratory Animal Co. Ltd. All experiments for C57BL/6 GF mice were performed in Shanghai SLAC Laboratory Animal Co. Ltd.

For the preparation of *huREG3*γ*^tgIEC^* mice, HD5 promoter, which may specifically promote the REG3γ expression in gut epithelial cells, was conjugated into Pinsulator–pHD5-promoter-CDS-poly plasmid. This conjugation was demonstrated by primers (REG3γ-HD5-tF: CATCCAACTCCAGGACGGAGTC and REG3γ-HD5-tR: CACCTGTAACATTGGCACTTTG) and sequencing using primers (Promoter-cx tF: GTTTGCTGGGTCAGAACTGA and Promoter-cx tR: GTAATTTAGGTGCGTACAGCC). Then the fragment of REG3γ-CDS and polyA were cloned into HD5 promoter-pinsulator. This conjugation was demonstrated using primers (REG3γ-PA-tF: CTTCCTGTGCAAGTACCGACC and REG3γ-PA-tR: GGTGTCTGCAGGCTCAAAGAG) and sequencing using the primers (HD5-Test-Tf: CTGTCTCAGGTCTTCTCCCAG and REG3γ -PA-F: GATCTTTTTCCCTCTGCCAAA). REG3γ transgenic mice were identified using the following primers (42: CTAGGCCACAGAATTGAAAGATCT and 43: GTAGGTGGAAATTCTAGCATCATCC, which produced 324 bp band). For experimental mice, WT mice first bred to *huREG3*γ*^tgIEC^* mice, and then *huREG3*γ*^tgIEC^* mice and their littermate WT mice were co-housed or separated into different cages after 4 weeks based on the experimental purpose.

All experimental litters were bred and maintained under specific pathogen-free (SPF) conditions in Nankai University. Experiments were carried out using age- and gender-matched mice. All the procedures were conducted according to the Institutional Animal Care and Use Committee of the Model Animal Research Center. Animal experiments were approved by the Institute’s Animal Ethics Committee of Nankai University. All experimental variables such as husbandry, parental genotypes, and environmental influences were carefully controlled. For high-fat diet-mediated obesity model, the experiments were strictly performed according to guidelines suggested by Stappenbeck and Virgin (20). Human samples were approved by the Institute’s Human Ethics Committee of Nankai University and agreed by individuals that offer fecal samples.

### Mouse Models

For microbiota transplantation, 6- to 8-week-old mice were treated with pan-antibiotics [ampicillin (A, 1 g/l, Sigma), vancomycin (V, 0.5 g/l), neomycin sulfate (N, 1 g/l), and metronidazole (M, 1 g/l)] *via* the drinking water. Water containing antibiotics was exchanged every 3 days. To confirm the elimination of bacteria, stool was collected from antibiotic-treated and untreated mice and cultured in anaerobic and aerobic conditions. The bacteria were counted under microscope. Then cecal contents from detergent treated mice or bacteria (1 × 10^9^) were suspended in 1 ml PBS with 30% glycerol. Mice (4 weeks old) were removed from the isolator and were orally administered 200 ml of fecal suspension or bacteria made using glycerol stocks.

For high-fat diet model, 6- to 8-week-old male mice and their control littermates were fed using high-fat diet (D12492, protein 26.2%, carbohydrate 26.3%, and fat 34.9%) and control diets (D12450B, 60% of calories may be derived from fat), which was from Research Diets, Inc. (NJ, USA).

For STAT3 inhibitor cucurbitacin I (JSI-124; Sigma Aldrich) treatment, pan-antibiotics-treated mice were infused with *Lactobacillus* NK318.1 (once/3 days, twice). Meanwhile, mice were treated ip with 1 mg/kg/of JSI-124 for 2 weeks (once/day).

For infusion of macrophages, mice before and after feeding high-fat diet were injected in tail vein using freshly isolated macrophages (1 × 10^6^/mice at day 0 and then once per week).

### Gut Microbiota

Gut microbiota was analyzed by Majorbio Biotechnology Company (Shanghai, China) using primers that target to the V3–V4 regions of the 16S rRNA. Once the PCR for each sample was completed, the amplicons were purified using the QIAquick PCR purification kit (Qiagen, Valencia, CA, USA), quantified, normalized, and then pooled in preparation for emulsion PCR followed by sequencing using Titanium chemistry (Roche, Basel Switzerland) according to the manufacturer’s protocol. In the first step of data processing, the generated sequence data were de-convolved using the sample barcodes to identify sequences from each of the samples. Barcode, primer, and adaptor sequences were also trimmed as part of this step. PCR artifacts “chimeras” were identified using the ChimeraSlayer program (http://microbiomeutil.sourceforge.net; reference http://genome.cshlp.org/content/21/3/494.long) and removed prior to downstream analysis. The resulting de-convoluted and filtered sequence data were assigned taxonomy (to the genus level) using the Ribosomal Database Project classifier, and the genera classifications were used to generate a sample genus count matrix. Operational Taxonomic Unit (OTU) analysis of these sequences was performed as follows: sequences were processed (trimmed) using the Mothur software and subsequently clustered at 97% sequence identity using cd-hit to generate OTUs. The OTU memberships of the sequences were used to construct a sample-OTU count matrix. The samples were clustered at genus and OTU levels using the sample genus and sample-OTU count matrices, respectively. For each clustering, Morisita-Horn dissimilarity was used to compute a sample distance matrix from the initial count matrix, and the distance matrix was subsequently used to generate a hierarchical clustering using Ward’s minimum variance method. The Wilcoxon Rank Sum test was used to identify OTUs that had differential abundance in the different sample groups. Bacterial DNA sequencing data have been uploaded to the Sequence Read Archive database in NCBI with accession number SAMN04569211 and SAMN04569229.

For absolute numbers of gut microbiota, 16S rRNAs were extracted and then amplified using phylum such as *Firmicutes, Bacteroidetes*, or genus-specific primers (Table S1 in Supplementary Material). The concentration of each product was detected and then exchanged into copy numbers. The purified products were diluted into 10^2^–10^8^ copies/ml and then analyzed by quantitative real-time PCR (qRT-PCR), and then standard curve was prepared based on the CT value and concentration. Other sample DNAs were amplified by qRT-PCR, and the exact copy number was counted based on the molecules. 100 mg of stool was suspended in 900 µl of cysteine–peptone–water solution and homogenized. For quantification of mucosa-associated bacteria, total DNA was isolated from PBS-washed and weighted gut tissue using DNeasy Blood & Tissue Kit (Qiagen). DNA was then subjected to quantitative PCR as described above, and results are expressed as bacteria number per milligram of gut tissue, using a standard curve. Primers used were listed in Table S1 in Supplementary Material.

### *Lactobacillus* Isolation and Culture

For lactobacillus isolation, 100 mg fresh fecal samples were collected and diluted in 2 ml BPS solution, cultured on Rogosa SL selective medium (Sigma Aldrich) for *Lactobacillus* enumeration, and then colonies were identified and purified for 16S ribosomal DNA sequence analyses for the speciation of colonial genotype. The lactobacilli were cultured in deMan, Rogosa, Sharpe (MRS; 3M Health Care, St. Paul, MN, USA) media and also grown on MRS agar containing either 10% sucrose. Anaerobic conditions were generated with sachets of AnaeroPack-Anaero (Mitsubishi Gas Chemical, Japan) in an air-tight jar. At 24 h of cultivation in liquid media, *Lactobacillus NK318.1* could reach 1 × 10^9^ CFU/ml.

### Immunostaining of Mucins and Localization of Bacteria by Fluorescent *In Situ* Hybridization

Mucus immunostaining was paired with fluorescent *in situ* hybridization (FISH) to analyze bacteria localization at the surface of the intestinal mucosa. In brief, ileum and colonic tissues (proximal colon, second centimeters from the cecum) containing fecal material were placed in methanol-Carnoy’s fixative solution (60% methanol, 30% chloroform, and 10% glacial acetic acid) for a minimum of 3 h at room temperature (RT). Tissue were then washed in methanol, ethanol, ethanol/xylene (1:1), and xylene, followed by embedding in paraffin with a vertical orientation. 5-mm sections were cut and dewaxed by preheating at 60°C for 10 min, followed by bathing in xylene at 60°C for 10 min, xylene at RT for 10 min, and 99.5% ethanol for 10 min. The hybridization step was performed at 50°C overnight with an EUB338 probe (59-GCTGCCTCCCGTAGGAGT-39 with a 5′ Cy3 label) diluted to a final concentration of 0.01 µg/ml in hybridization buffer (20 mM Tris–HCl, pH7.4, 0.9 M NaCl, 0.1% SDS, 20% formamide).

For *Lactobacillus*, the hybridization step was performed at 60°C overnight with an RNA probes (Lac663: FAM-O-ACATGGAGTTCCACT) diluted to a final concentration of 0.01 µg/ml in hybridization buffer [10% dextran sulfate (wt/vol), 10 mM NaCL, 30% (vol/vol) formamide, 0.1% (wt/vol) Ficoll, 5 mM Na_2_EDTA, 0.1% (wt/vol) SDS, 0.1% (vol/vol)triton X-100, 50 mM Tris–HCl, pH = 7.4]. After washing for 10 min in wash buffer (wash solution: 2× SSC, 0.1% Tween-20) and 10 min in PBS, block solution (5% FBS in PBS) was added for 30 min at 50°C. Mucin 2 primary antibody (rabbit H300, Santa) was diluted to 1:1,500 in block solution and applied overnight at 4°C. After washing in PBS, block solution containing anti-rabbit secondary antibody diluted to 1:1,500 was applied to the section for 2 h. After washing in PBS, slides were mounted using Prolong anti-fade mounting media (Life Technologies). Nuclei were stained using Hoechst 33342. Observations were performed with a Zeiss LSM 700 confocal microscope with software Zen 2011 version 7.1. This software was used to determine the distance between bacteria and the epithelial cell monolayer, as well as the mucus thickness.

#### Microarray

Total RNA was extracted using Trizol (Life technologies, Carlsbed, CA, USA) according to the manufacturer’s instructions. Contaminating DNA was removed using RNeasy spin columns (Qiagen, Valencia, CA, USA). The quality of isolated RNA samples was evaluated with an Agilent Bioanalyzer 2100 (Agilent technologies, Santa Clara, CA, USA), and the purified RNA was quantified using a NanoDrop ND-1000 spectrophotometer (Infinigen Biotechnology Inc., City of Industry, CA, USA). An Affymetrix GeneChip Hybridization Oven 640 and Gene Array Scanner 3000 were used to perform microarray analysis. The R software (v.2.13.0) platform was applied to analyze the microarray data, and the limma (linear regression model) package was used to statistically analyze differentially expressed genes (21, 22). The expression levels of mRNAs at each time point were compared with control. Genes having a fold change >2 or <−2 and an adjusted *p* < 0.05 were considered as differentially expressed.

For bioinformatic analysis, bioinformatic analysis tools, including Euclidean distance calculation, hierarchical clustering, PCA, GO, KEGG, and IPA, were applied to investigate differentially expressed genes PNI. Briefly, Euclidean distance calculation was performed using the HeatMapImage GenePattern module, and hierarchical clustering was computed with the Euclidean distance measure using the hierarchical clustering module from GenePattern. PCA was performed using “Population PCA” tool from Harvard Medical School. Database for Annotation, Visualization, and Integrated Discovery bioinformatic resources were used to systematically screen differentially expressed genes and to enrich significant GO categories and KEGG pathways. IPA was performed to identify and connect differentially expressed transcription factors. For microarray GEO accession number GSE98819, see https://www.ncbi.nlm.nih.gov/geo/query/acc.cgi?acc=GSE98819.

#### Macrophage Functions

For arginase activity, cells were lysed for 30 min with 100 µl of 0.1% Triton X-100 at 4°C. Following lysis, 100 µl of 25 mM Tris–HCl and 10 µl of 10 mM MnCl_2_ were added, and the mixture was heated for 10 min at 56°C. Subsequently, the lysates were incubated with 100 µl of 0.5 M l-arginine (pH 9.7) at 37°C for 120 min. The reaction was stopped with 900 µl of H_2_SO_4_ (96%)/H_3_PO_4_ (85%)/H_2_O (1:3:7). Urea concentration was measured by absorbance at 540 nm after addition of 40 µl of 9% α-isonitrosopropiophenone, followed by heating at 95°C for 30 min. A standard curve was generated by serial dilution of 120 mg/ml urea. Arginase activity (Unit) was defined by the amount enzyme that catalyzes the formation of 1 μg of urea per minute.

For nitric oxide production, nitric oxide in culture supernatant was assayed using Griess Reagent System. Equal volumes of supernatant (50 µl) and sulfanilamide solution (1% sulfanilamide in 5% phosphoric acid) were incubated at RT for 5–10 min, followed by addition of 50 μl of NED solution (0.1% *N*-1-napthylethylenediamine dihydrochloride). After incubation for 10 min at RT, absorbance at 550 nm was measured. Nitrite concentrations were quantified by comparing the absorbance values to a standard curve generated by serial dilution of 100 μM sodium nitrite.

For H_2_O_2_ production, production of H_2_O_2_ was evaluated using Amplex Red Hydrogen Peroxide/Peroxidase Assay Kit (Invitrogen). Briefly, 1 × 10^4^ cells were resuspended in Hanks Balanced Salt solution. After addition of phorbol 12-myristate 13-acetate (PMA) (30 ng/ml), the absorbance at 560 nm was measured using a microplate reader at 37°C. Absorbance values for the test samples were normalized to a standard curve generated by serial dilutions of 10 mM H_2_O_2_.

For T cell suppression assay, round bottom 96-well plates were coated with anti-CD3/CD28 antibody (BD Pharmingen, CA, USA) in PBS at 0.5 µg/ml at 4°C overnight. 1 × 10^6^ CD4^+^ or CD8^+^ T cells were purified from normal splenocytes with microbeads and stained with 10 µM CFSE (Invitrogen, Carlsbad, CA, USA) for 1 min at 37°C and co-cultured with 5 × 10^4^ isolated macrophages for 3 days in anti-CD3/CD28 antibody-coated 96-well plates at 1 × 10^6^ cells/ml in the presence of 2 ng/ml IL-2 with RPMI 1640 medium, containing 5% FBS, 100 IU/ml penicillin 100 μg/ml streptomycin. CFSE-stained CD4 or CD8 T cells were analyzed at channel 1 (FL1).

For CD3-ζ Chain staining, T cell surface marker (CD4 and/or CD8) staining was performed prior to fixation, and then cells were washed with PBS twice, followed by fixed with 1% paraformaldehyde in PBS for 15 min, permeabilized with 0.1% Triton X-100 for 10 min, and then washed in PBS. The fixed and permeabilized cells were incubated in 2% BSA/PBS for 30 min to block non-specific binding. After washing, the cells were incubated with anti-CD3-ζ mAb for 1 h. All steps were performed at 4°C. Cells were analyzed on a FACScan flow cytometer.

For intracellular signal inhibition, freshly isolated macrophages were exposed to heated-dead *Lactobacillus NK318.1* with NFκB inhibitor (QNZ, 20 nM), JNK inhibitor (JNK-IN-8, 1 μM), ERK inhibitor (SCH772984, 10 nM), and STAT3 inhibitor cucurbitacin I (JSI-124, 3 µM), respectively.

#### Flow Cytometry

Single-cell suspensions of mesenteric lymph nodes (MLNs), Peyer’s patches (PP), and spleen of mice were prepared by mashing in a cell strainer (70 mm), stained, and analyzed by flow cytometry. For the staining of LP lymphocytes, gut epithelial cells were first removed away using 5 mM EDTA and then digested in RPMI 1640 with 5% FBS and 1 mg/ml collagenase IV and 1 mg/ml dispase (Invitrogen) for 1 h at 37°C. LP cells were filtered to minimize mucus contamination and purified using Percoll gradient, then stained, and analyzed by flow cytometry. Dead cells were eliminated through 7-AAD staining.

For intracellular staining, the cells were cultured and stimulated for 16 h with 50 ng/ml PMA (Sigma) and 1 µg/ml ionomycin (Sigma) in the presence of GolgiStop (10 ng/ml, BD Biosciences). After incubation for 16 h, cells were washed in PBS, fixed in Cytofix/Cytoperm, permeabilized with Perm/Wash buffer (BD Biosciences), and stained with FITC-, PE-, APC- APC/cy7-, PerCP/Cy5.5-, or PE/cy7-conjugated antibodies.

Except special indication, the cells from LP, PP, MLN, and adipose tissues were gated according to gated strategy in Figure S2 in Supplementary Material.

#### Immunostaining

5-µm-thick sections were prepared from the frozen tissue and fixed in acetone (−20°C) for 10 min. After rehydration in PBS for 5 min and further washing in PBS, tissue sections were blocked with 1% (w/v) BSA and 0.2% (w/v) milk powder in PBS (PBS-BB). The primary antibody was added in PBS-BB and incubated overnight at 4°C. After PBS washing (three times, 5 min each), tissue was detected with DAB kit or fluorescence-labeled second antibody. Nuclei were stained by DAPI.

#### Detection of IL-10, Arginase-1, H_2_O_2_, and NO

Gut tissue was milled in liquid nitrogen and then lysed with non-denatured tissue lysis solution (Cat#: R0030, Solarbio, Beijing, China). 50 µl lysate was subjected to IL-10, arginase-1, H_2_O_2_, and NO detection with mouse IL-10 ELISA kit (Cat#: CME0016, 4A biotech, Beijing China), arginase-1, H_2_O_2_, and NO Kit.

#### Metabolism Experiments

For glucose and insulin tolerance, after 5 h fasting, baseline blood glucose levels were measured using a Nova Max Plus GlucoseMeter. Mice were then injected intraperitoneally with 2 g glucose per kilogram body weight in sterile PBS or with 0.5 U insulin per kilogram body weight (Sigma, St. Louis, MO, USA), and blood glucose levels were measured 30, 60, 90, and 120 min after injection.

Serum glucose (Glu), cholesterol (CHO), and triglyceride (TG) were analyzed using blood biochemical Analyzer (GRT-3006).

#### Western Blotting

Cell lysates were denatured and subjected to SDS-PAGE and then were transferred to PVDF membranes. The membranes were incubated with the primary antibody, followed by hybridization with the secondary HRP-conjugated antibody. Detection was performed using an enhanced chemiluminescence assay with Lumi-Glo reagents (Millipore).

#### RT-PCR and qRT-PCR

Total RNA was extracted from the cells, tissues, and organs using TRIzol reagent (Invitrogen). First-strand cDNA was generated from total RNA using oligo-dT primers and reverse transcriptase (Invitrogen Corp). The PCR products were visualized on 1.0% (wt/vol) agarose gels. qRT-PCR was conducted using QuantiTect SYBR Green PCR Master Mix (Qiagen) and specific primers in an ABI Prism 7000 analyzer (Applied Biosystems). GAPDH mRNA expression was detected in each experimental sample as an endogenous control. All the reactions were run in triplicate. The primers used in this study were listed in Table S1 in Supplementary Material.

### Statistical Analysis

Student’s *t*-test, one-way analysis of variance, and the Mann–Whitney U test were used to determine significance. A 95% confidence interval was considered significant and was defined as *p* < 0.05. *p < 0.05, **p < 0.01, and ***p < 0.001.

## Results

### REG3γ Overexpression Causes Altered Composition of Gut Microbiota and Enlarged Macrophage Pools

**Figure 1:**
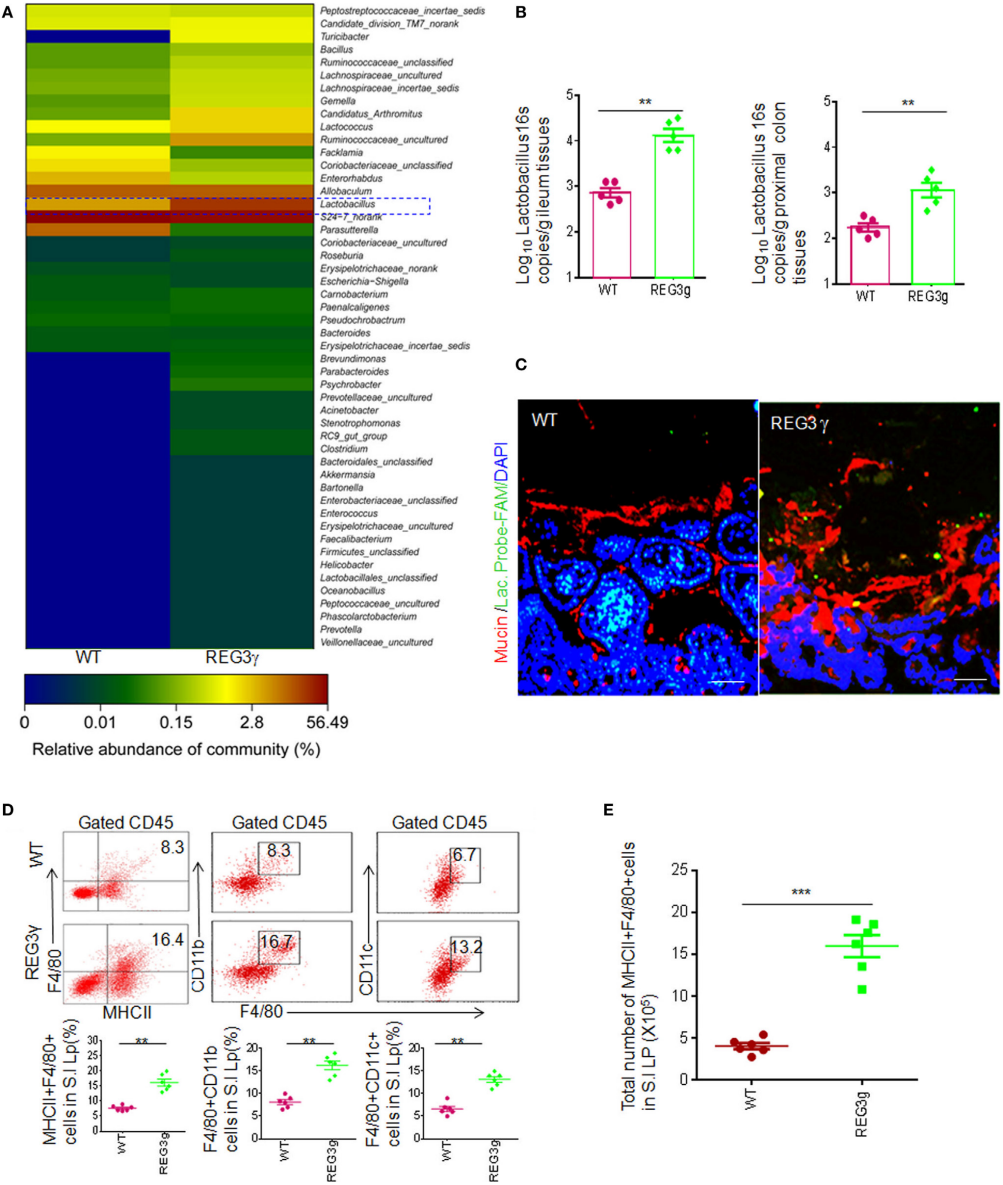

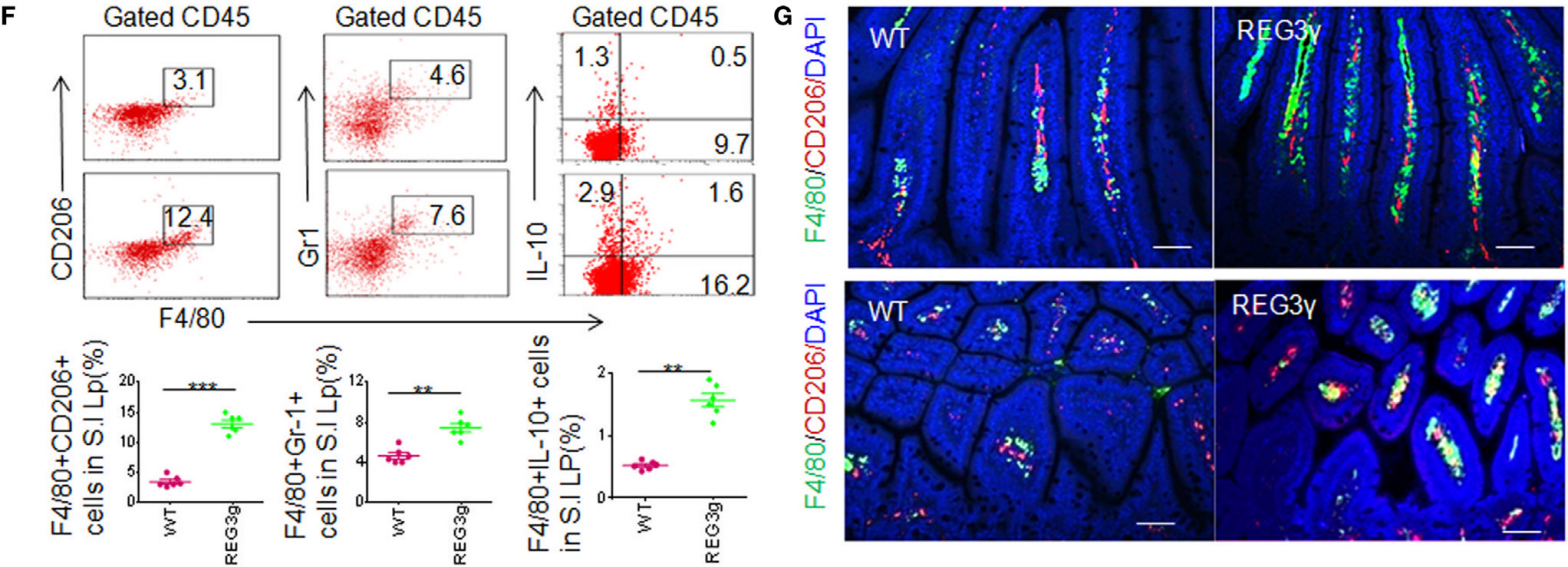
REG3γ overexpression causes alterations of gut microbiota and expands the pools of anti-inflammatory macrophages. **(A)** 16S rRNA analyses of gut microbiota in *huREG3*γ*^tgIEC^* and control littermate WT mice. The weaned *huREG3*γ*^tgIEC^* (REG3γ) and control littermate WT mice were reared in different cages, and then 16S rRNAs from pooled small intestinal content samples (*n* = 5, 7- to 8-week-old male mice fed normal chow) were amplified using primers that targeted the V3–V4 regions of the 16S rRNA. The samples were clustered at genus levels using the sample genus count matrices. WT, wild-type; REG3γ, *huREG3*γ*^tgIEC^* mice. See also NCBI with accession number SAMN04569211 and SAMN04569229. Data are a representative of three independent experiments. **(B)** Q-PCR of *Lactobacillus* genus in the ileum and proximal colon tissues of WT and *huREG3*γ*^tgIEC^* mice fed normal chow (*n* = 5). Standard curves were prepared from serial dilution of *Escherichia coli* genomic 16S rRNA. **(C)** Fluorescence *in situ* hybridization and immunostaining of ileum fragments in 7- to 8-week-old male *huREG3*γ*^tgIEC^* mice (REG3γ) and control littermate WT mice. The representative images from five mice per group; Scale bars = 40 μm. **(D)** Flow cytometry of MHCII(+)F4/80(+), F4/80(+)CD11b(+), and F4/80(+)CD11C(+) macrophages in small intestinal (S.I) lamina propria of *huREG3*γ*^tgIEC^* (REG3γ/REG3g) and control littermate WT mice. The proportion of MHCII(+)F4/80(+) macrophages in the S.I lamina propria of WT and *huREG3*γ*^tgIEC^ mice* were compared (*n* = 6; lower). **(E)** Comparison of total MHCII(+)F4/80(+) macrophages in the whole S.I lamina propria of *huREG3*γ*^tgIEC^* (REG3g) and control littermate WT mice (*n* = 6). **(F)** Flow cytometry of F4/80(+)CD206(+), F4/80(+)Gr-1(+), and F4/80(+)IL-10(+) macrophages in the S.I lamina propria of *huREG3*γ*^tgIEC^* and control littermate WT mice. The proportion of MHCII(+)F4/80(+), F4/80(+)Gr-1(+), and F4/80(+)IL-10(+) macrophages in WT and *huREG3*γ*^tgIEC^* (REG3g) mice were compared (*n* = 6). **(G)** Immunostaining of F4/80(+)CD206(+) macrophages in the ileum tissues of *huREG3*γ*^tgIEC^* (REG3γ) and control littermate WT mice. The representative images from six mice per group; scale bars = 40 μm. *p < 0.05, **p < 0.01, and ***p < 0.001 (*t*-test, mean ± SD); NS, no significant. See also Figures S1–S3 in Supplementary Material.

Gut epithelial cells that produce bacteria-killing substance may affect the composition of gut microbiota (23, 24). REG3γ, a kind of bacteria-killing substance, may target Gram-positive bacteria (18). However, *Reg3*γ^−/−^ mice do not exhibit significant alterations in gut luminal bacterial communities (25). Interestingly, in *Reg3*γ*^tgIEC^* mice, there exist significant differences in the composition of gut microbiota not only in gut mucus layer but also in luminal microbiota (24). Thus, REG3γ overexpressing mice offer a model to found some bacteria, which may potentially affect gut immune cell populations. We generated gut epithelial cell-restricted human REG3γ transgenic (*huREG3*γ*^tgIEC^*) mice. Human REG3γ (Gene ID: 130120) is selectively expressed in mouse gut epithelial Paneth cells under the HD5 promoter (26). Consistent with other data (25), REG3γ could increase spatial space to segregate bacteria from gut epithelial cells. We compared the composition of gut microbiota of *huREG3*γ*^tgIEC^* mice and their WT littermate mice by sequencing 16S rRNAs (V3–V4 regions) in pooled small intestine contents in male WT and *huREG3*γ*^tgIEC^* mice and observed dramatic changes of the gut microbiota in mice fed with standard chow (Figure 1A). Although the antibacterial activity of REG3γ is restricted to Gram-positive bacteria (18), the proportion of *Lactobacillus genus* (Gram positive) was higher in *huREG3*γ*^tgIEC^* mice than those in WT mice, consistent with the results from mouse *Reg3*γ*^tgIEC^* mice (24). The high proportion of *Lactobacillus genus* in REG3γ overexpressing mice may be derived from the resistance of healthy microbiota (27). Notably, increased *Lactobacilli* were also detected not only in mucus layers of small intestine but also in colonic tissues by using q-PCR and fluorescence *in situ* hybridizations (Figures 1B,C). These results suggest that REG3γ overexpression in gut epithelial cells may affect the composition of gut microbiota.

Alterations of gut microbiota may affect the composition of gut immune cells and immune cell subsets (1, 14–16). Since *Lactobacilli* are rich in small intestine mucus layers of *huREG3*γ*^tgIEC^* mice, we next investigated the composition of gut immune cells in the LP of ileum tissues using flow cytometry based on the gating strategies (Figure S1A). There was remarkably increased proportion of MHCII(+)F4/80(+) macrophages in the ileum LP in *huREG3*γ*^tgIEC^* mice compared to control WT littermates (Figure 1D). Similar to previous data in WT mice (6), these MHCII(+)F4/80(+) macrophages in the *huREG3*γ*^tgIEC^mice* could also express CD11c and CD11b (Figure 1D). The absolute number of MHCII(+)F4/80(+) macrophages was remarkably higher in the small intestine of *huREG3*γ*^tgIEC^* mice compared to control WT (Figure 1E). However, these MHCII(+)F4/80(+) cells also included CD206(+) and CD206(−), Gr-1(+) and Gr-1(−), and IL-10(+) and IL-10(−) macrophage subpopulations (Figures 1F,G). Furthermore, the proportion of MHCII(+)F4/80(+)CD206(+), MHCII(+)F4/80(+)Gr-1(+), and MHCII(+)F4/80(+)IL-10(+) macrophage subsets also increased in the LP of *huREG3*γ*^tgIEC^* mice (Figures 1F,G). Notably, CD11b(+)Gr-1(+) myeloid-derived suppressive cells (MDSCs) and MHCII(+)CD11C(+) cells were much more in the LP of *huREG3*γ*^tgIEC^* mice in different degrees. The proportion of Treg cell subsets also increased in *huREG3*γ*^tgIEC^mice* (Figure S2A in Supplementary Material), which may be related to enlarged anti-inflammatory macrophage pools (28). IL-10 levels in the ileum tissues of *huREG3*γ*^tgIEC^* mice, which is related to anti-inflammatory macrophages and Treg, were higher than WT mice (Figure S2B in Supplementary Material). The proportion of other immune cell populations and subpopulations such as B cells, CD4, CD8, and other T cell subpopulations such as Th1, Th17, and IL-22 cells did not exhibit significant changes. These data indicate that REG3γ may specifically affect the differentiation of myeloid-derived cells such as macrophages.

Gut immune cells have been shown to possess broad trafficking abilities to lymphoid organs at distant sites, such as the MLN and spleen (29). Increased F4/80(+)MHCII(+) macrophages and macrophage subpopulations were also detected in the PP, MLN, and spleen of *huREG3*γ*^tgIEC^* mice (Figures S2C,D in Supplementary Material). Notably, those increased subsets MHCII(+)F4/80(+)CD206(+), MHCII(+)F4/80(+)Gr-1(+), and MHCII(+)F4/80(+)IL-10(+) in the LP were also found in the PP and spleen of *huREG3*γ*^tgIEC^* mice (Figures S2E,F in Supplementary Material). Taken together, our data demonstrate that REG3γ overexpression enlarges macrophage pools not only in the LP but also in the PP, MLN, and spleen.

Since MHCII(+) and F4/80(+) are makers of mature, anti-inflammatory macrophages in gut tissues (9), we also assessed immune tolerance situation at basic line. Beyond increased Treg cells and IL-10 in the LP of ileum tissues, myeloid cell-derived immune suppressor substance arginase-1, NO, and H_2_O_2_ were also higher in the LP of *huREG3*γ*^tgIEC^* mice compared to control WT (Figure S3A in Supplementary Material). The expression levels of CD3 zeta chain of CD4 and CD8 cells in the LP of *huREG3*γ*^tgIEC^* mice were significantly lower in *huREG3*γ*^tgIEC^* mice (Figure S3B in Supplementary Material). The levels of inflammation cytokines including TNFα, IL-6, IL-1β, and IFNγ were lower in *huREG3*γ*^tgIEC^* mice compared to control WT littermate mice (Figure S3C in Supplementary Material). Compared to control WT, freshly isolated MHCII(+)F4/80(+) macrophages (Figure S1B in Supplementary Material) from *huREG3*γ*^tgIEC^* mice could express higher levels of IL-10 and higher levels of arginase-1, NO, and H_2_O_2_ (Figure S3D in Supplementary Material). These IL-10, NO, and H_2_O_2_ expression was further augmented by exposure to *Lactobacillus NK318.1* (Figure S3E in Supplementary Material). Freshly isolated macrophages from the LP of *huREG3*γ*^tgIEC^* mice also exhibited stronger suppression on CD4 and CD8 T cell proliferation (Figure S3F in Supplementary Material). Thus, our data suggest that increased macrophages in *huREG3*γ*^tgIEC^* mice promote the homeostasis of gut tissues.

### Gut REG3γ-Associated *Lactobacillus NK318.1* Alone Expand Anti-inflammatory Macrophage Pools

Next, we investigated how REG3γ cause the accumulation of anti-inflammatory macrophages. Previous studies showed that the composition of gut microbiota may affect the differentiation of gut immune cell populations or their subpopulations (1, 14–16). The proportion of macrophages in pan-antibiotics (ampicillin, vancomycin, neomycin sulfate, and metronidazole)-treated *huREG3*γ*^tgIEC^* mice were remarkably deceased (not shown), implying that gut microbes are involved in the increased macrophages in *huREG3*γ*^tgIEC^* mice. Since the proportion of *Lactobacillus* was remarkably increased among the composition of gut microbiota in *huREG3*γ*^tgIEC^* mice, we hypothesized that these increased *Lactobacilli* might be related to the increased macrophages. One *Lactobacillus* strain named as *Lactobacillus NK318.1*, which is close to *Lactobacillus Taiwanesis* isolate (Figures S4A,B in Supplementary Material), was very often detected in the isolated *Lactobacillus* clones using *Lactobacillus* selective medium (Rogosa SL selective medium) from the fresh feces of *huREG3*γ*^tgIEC^* mice (35% in *huREG3*γ*^tgIEC^* mice, whereas 5% in WT mice). 16S rRNA copies of *Lactobacillus NK318.1* were also much more in the fresh feces of *huREG3*γ*^tgIEC^* mice compared to control WT littermates (Figure S4C in Supplementary Material). Other *Lactobacillus* such as *L. reuteri, Lactobacillus gasseri, Lactobacillus animalis, Lactobacillus rhamnosus*, and *Lactobacillus plantarum* did not significantly change and were even decreased in *huREG3*γ*^tgIEC^* mice (Figure S4C in Supplementary Material). While equal amounts of *Lactobacillus NK318.1, Lactobacillus NK318.2* with high homology to*L. animalis* (Figures S4D,E in Supplementary Material), *L. reuteri* and positive control *huREG3*γ*^tgIEC^* mouse feces were transplanted into antibiotics-treated mice by orally gavage (Figure S5A,B in Supplementary Material), increased MHCII(+)F4/80(+) and F4/80(+)IL-10(+) macrophages were only detected in the *Lactobacillus NK318.1*-transplanted mice, whereas *Lactobacillus NK318.2* and *L. reuteri* did not cause similar changes (Figures S5C–E in Supplementary Material). IL-10 levels in ileum tissues and sera of in the *Lactobacillus NK318.1*-transplanted mice were higher compared to control untransplanted mice (Figure S5F in Supplementary Material). All of these suggest that *Lactobacillus NK318.1* may be a bacterium, which may enlarge gut anti-inflammatory macrophage pools.

**Figure 2:**
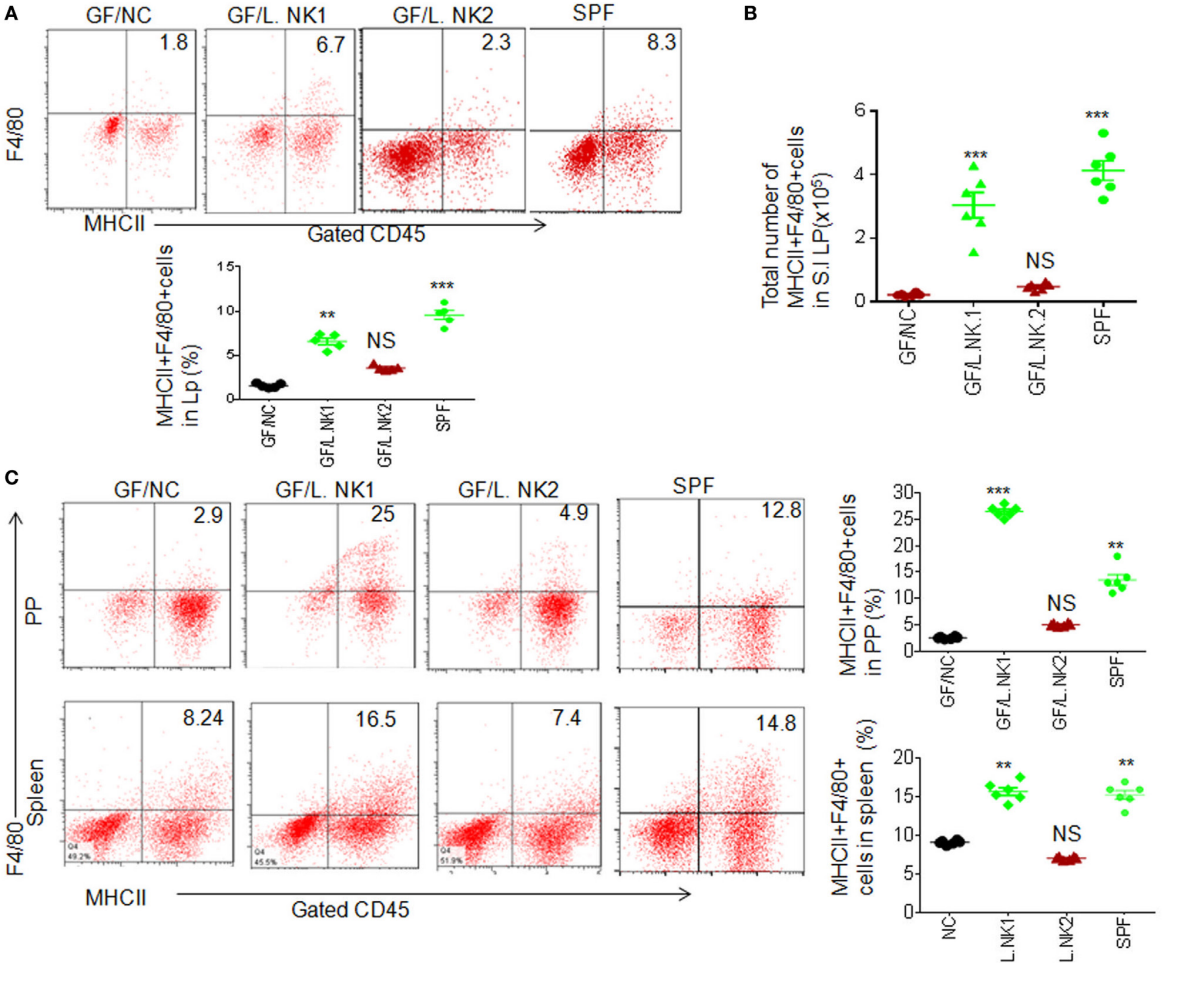

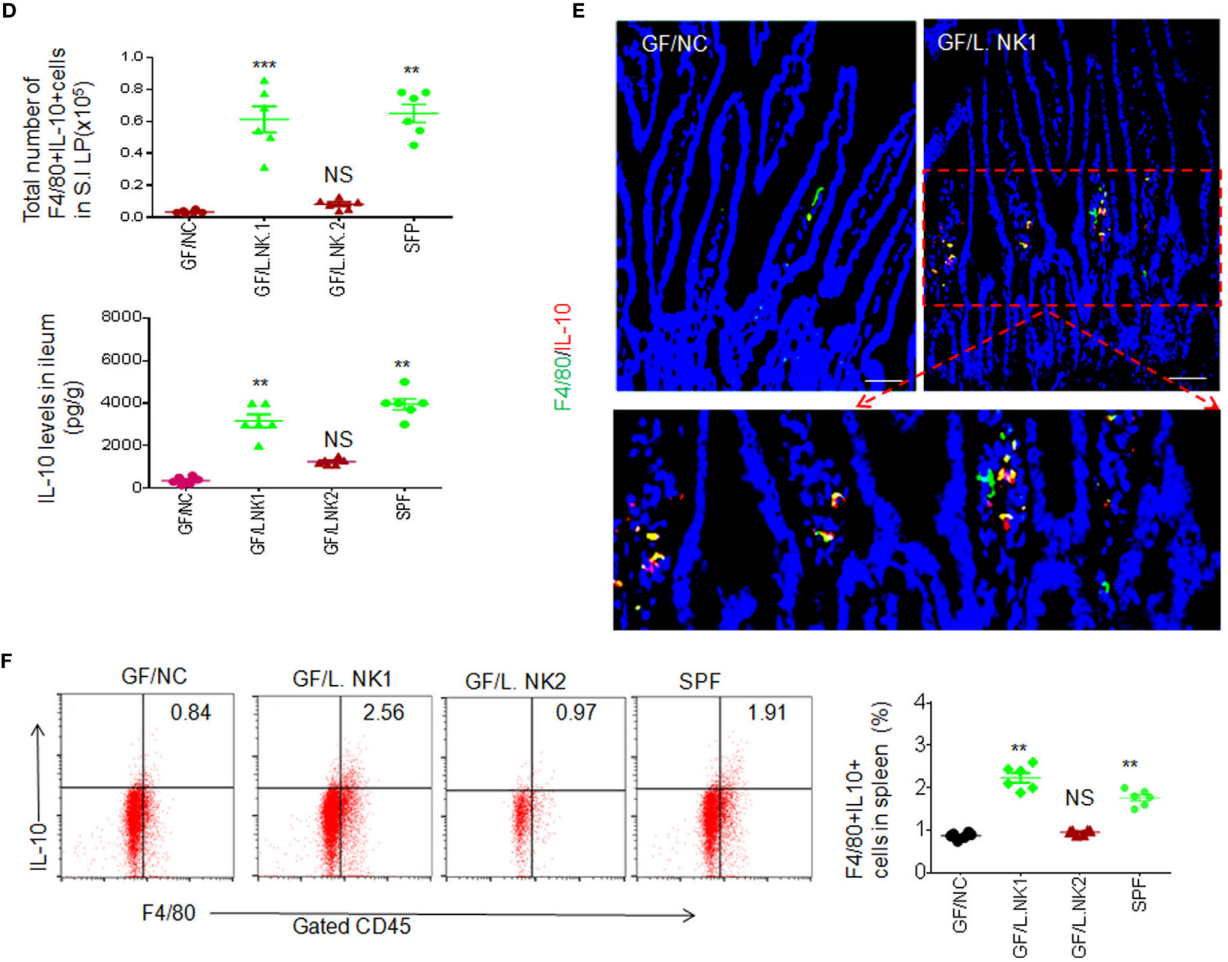
REG3γ-associated *lactobacillus* induces accumulation of macrophages in germ-free (GF) mice. **(A)** Flow cytometry of macrophages in small intestinal lamina propria (LP) of GF mice with or without L.NK1 or L.NK2 colonization. The proportion of MHCII(+)F4/80(+) macrophages in GF mice with (GF/L.NK1 or GF/L.NK2) and without (GF/NC) *Lactobacillus* colonization were compared (lower; *n* = 6). **(B)** Comparison of total MHCII(+)F4/80(+) macrophages in whole small intestinal LP of GF mice with (GF/L.NK.1 or GF/L.NK2) and without (GF/NC) *Lactobacillus* colonization (*n* = 6). **(C)** Flow cytometry of MHCII(+)F4/80(+) macrophages in the PP and spleen of L.NK1 (GF/L.NK1) or L.NK2 (GF/L.NK2) colonized GF mice. The proportion of MHCII(+)F4/80(+) macrophages in GF mice with (GF/L.NK.1 or GF/L.NK2) and without (GF/NC) *Lactobacillus* colonization were compared (right; *n* = 6). **(D)** Comparison of total F4/80(+)IL-10(+) macrophages (upper) and interleukin (IL)-10 levels (lower) in the whole small intestinal LP of GF mice with (GF/L.NK.1 or GF/L.NK2) or without (GF/NC) *Lactobacillus* colonization (*n* = 6). **(E)** Immunostaining of F4/80(+)IL-10(+) macrophages in the ileum tissues with or without L.NK.1 colonization. The representative images of six mice per group; scale bars = 40 μm. **(F)** Flow cytometry of F4/80(+)IL-10(+) macrophages in the spleen of L.NK.1 (GF/L.NK.1) or L.NK.2 (GF/L.NK2) colonized GF mice. The proportion of F4/80(+)IL-10(+) macrophages in GF mice with (GF/L.NK.1 or GF/L.NK2) and without (GF/NC) *Lactobacillus* colonization were compared (right; *n* = 6). Specific pathogen-free (SPF), wt mice raised in SPF environment; *p < 0.05, **p < 0.01, and ***p < 0.001 (one-way analysis of variance, mean ± SD). See also Figures S4 and S5 in Supplementary Material.

To determine whether anti-inflammatory macrophages are really related to REG3γ-associated *Lactobacillus NK318.1*, we next colonized GF mice with *Lactobacillus NK318.1*. After 2 weeks, *Lactobacillus* colonization were demonstrated through FISH analyses with a specific probe. Consistent with other data (9), non-transplanted control GF mice housed under separate but similar conditions had very few macrophages (Figures 2A,B). In contrast, *Lactobacillus*-transplant induced robust accumulation of F4/80 macrophages not only in the LP of ileum tissues but also in the PP and spleen (Figure 2C). Moreover, the effect of *Lactobacillus* is bacterial species specific. *Lactobacillus NK318.1* but not *Lactobacillus NK318.2* could cause accumulation of MHCII(+)F4/80(+) macrophages in the gut LP, PP, and also spleen of GF mice (Figures 2A,C). The proportion of IL-10(+) macrophages was remarkably increased in the LP, PP, and spleen of *huREG3*γ*^tgIEC^* mice (Figures 2D–F; not shown). The levels of IL-10 were also higher in the gut tissues of the LP in *Lactobacillus NK318.1*-transplanted GF mice (Figure 2D). Increased IL-10 (+) macrophages in the ileum LP were also further confirmed by immunohistochemistry staining (Figure 2E). In addition, transplantation of *Lactobacillus NK318.1* could reverse many intestinal immune abnormalities, including prolonging the length of small intestine and colon and promoting the establishment and mature of gut immune system such as remarkably larger PP and expanded immune cells in the *Lactobacillus*-transplanted mice, which are similar to SPF mice. Taken together, our data show that REG3γ-associated *Lactobacillus* may enlarge MHCII (+)F4/80(+) macrophage pools not only in the LP but also in the PP and spleen.

### STAT3 Is Involved in *Lactobacillus NK318.1*-Mediated Accumulation of Macrophages

**Figure 3:**
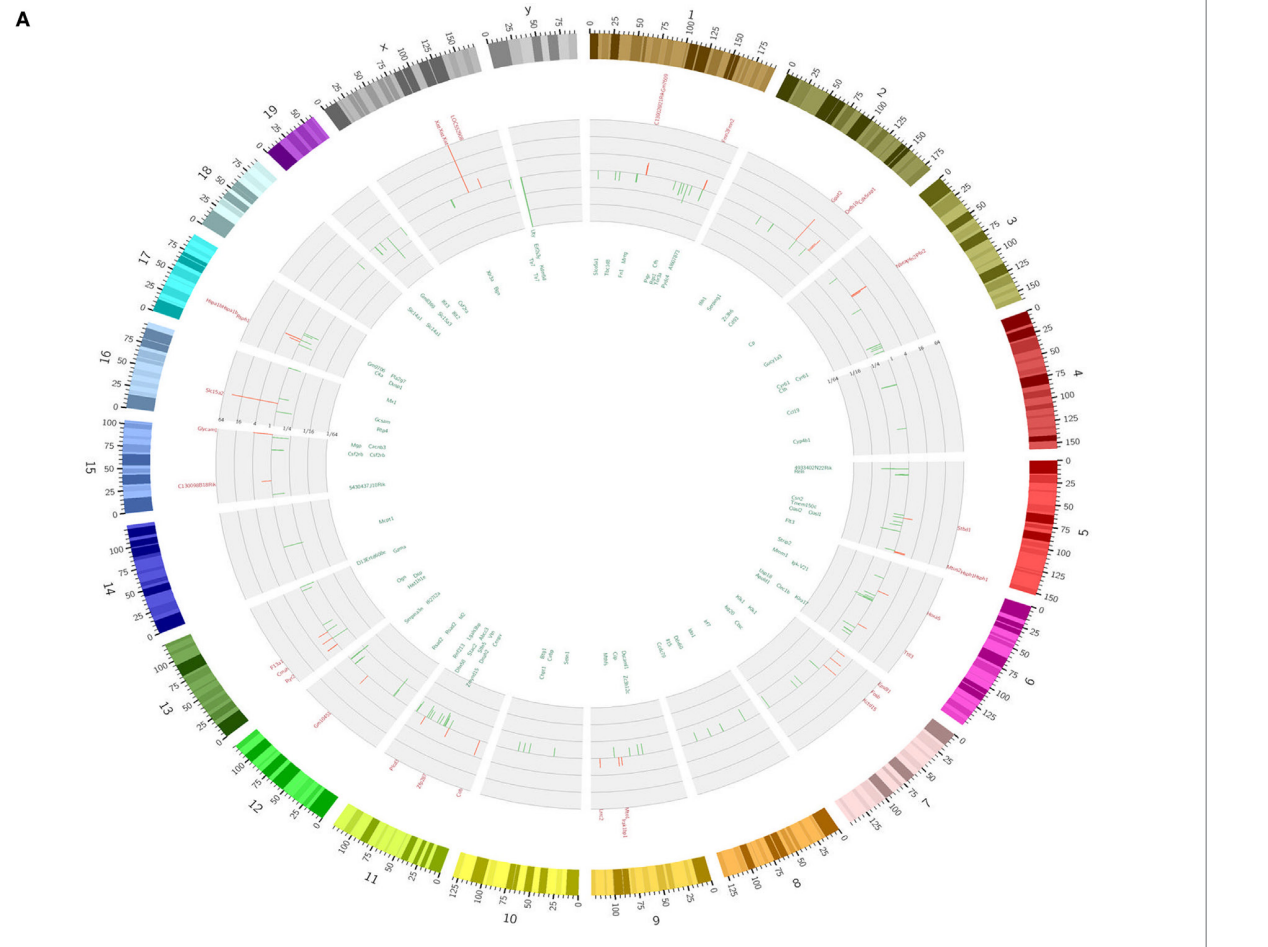

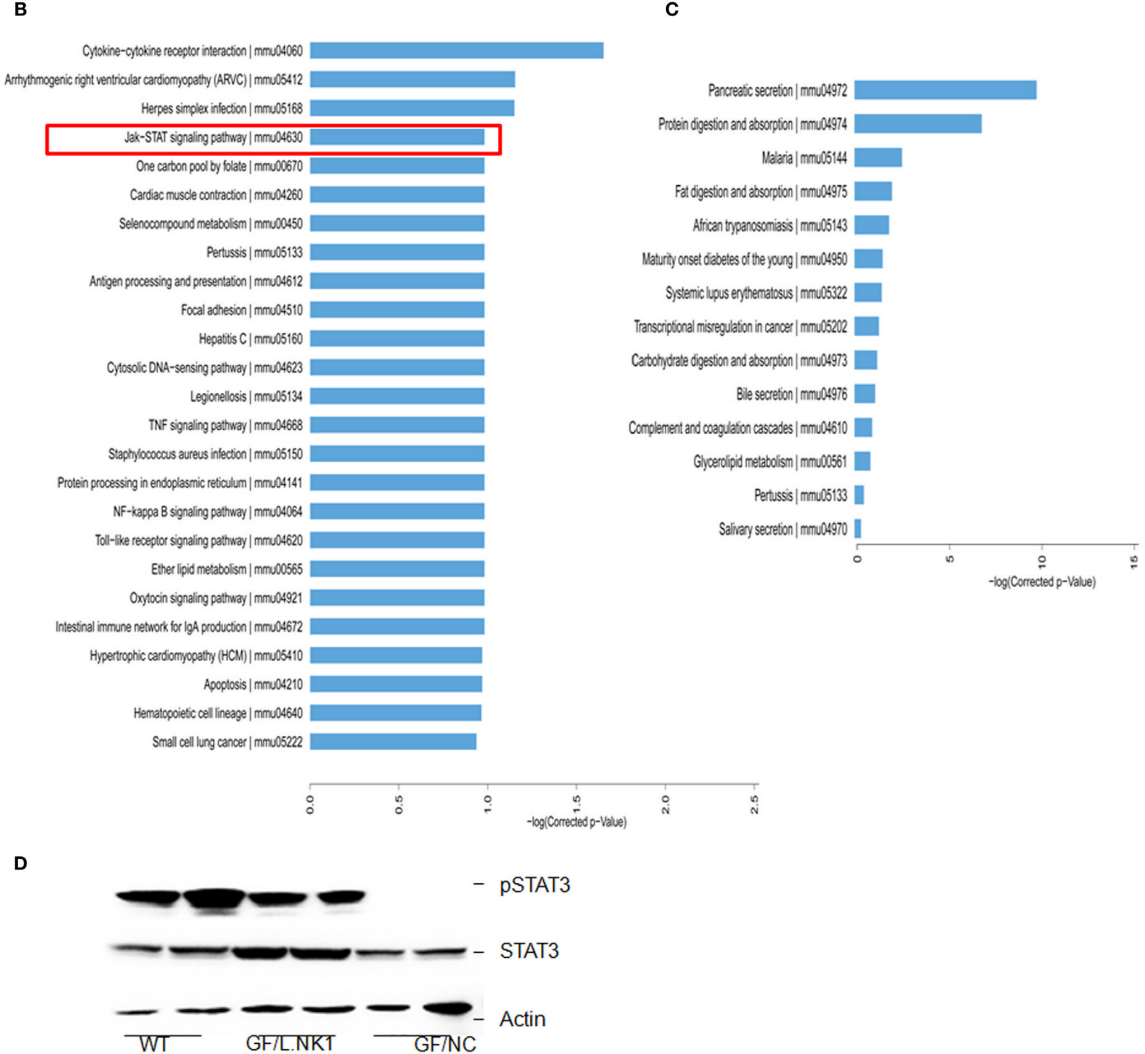
STAT3 is activated in the gut immune tissues of *lactobacillus NK318.1*-colonized GF mice. **(A)** Circos analyses of gene expression in the Payer’s patch of GF mice with (L.NK.1) or without *Lactobacillus NK318.1* (L.NC) colonization (pooled samples; *n* = 6). See also microarray GEO accession number GSE98819: https://www.ncbi.nlm.nih.gov/geo/query/acc.cgi?acc=GSE98819. **(B)** Enriched KEGG pathways of differentially expressed genes in the Payer’s patch of GF mice with (L.NK.1) or without *Lactobacillus NK318.1* (L. NC) colonization (pooled samples; *n* = 6). **(C)** Enriched KEGG pathways of differentially expressed genes in the ileum epithelial cells of GF mice with (L.NK.1)or without *Lactobacillus NK318.1* (L. NC) colonization (pooled samples; *n* = 6). **(D)** Immunoblotting of phosphor-STAT3 in the ileum lamina propria tissues of GF mice with (GF/L.NK1) or without (GF/NC) *Lactobacillus NK318.1* colonization. WT, wild-type; GF, germ free.

To found a mechanism for how *Lactobacillus* causes increased macrophages, we first compared the gene expression profiles in the Payer’s patch (aggregated lymphoid nodules in gut LP) in GF mice with or without *Lactobacillus* colonization. *Lactobacillus NK318.1* colonization in GF mice might induce at least a twofold change in expression of multiple gene expression (Figure 3A). KEGG analyses on those higher expression genes, which are induced by *Lactobacillus*, identified several critical signaling pathways, including Janus-activated kinase (JAK)-STAT signaling pathway, Toll-like receptor signaling pathway, NF-κB signaling pathway, TNF signaling pathway, intestinal immune network for IgA production, and cytokine–cytokine receptor interaction (Figure 3B). Notably, this strain of *Lactobacillus* did not affect immune-associated signaling pathways in gut epithelial cells (Figure 3C), indicating that *Lactobacillus NK318.1*-mediated accumulation of macrophages were not through gut epithelial cells. Activated STAT3 were further detected in the freshly gut tissues of *Lactobacillus* colonized GF mice through immunoblotting but not in GF mice without *Lactobacillus* colonization (Figure 3D). These data suggest that STAT3 may be involved in the *Lactobacillus*-mediated accumulation of macrophages in gut tissues.

**Figure 4:**
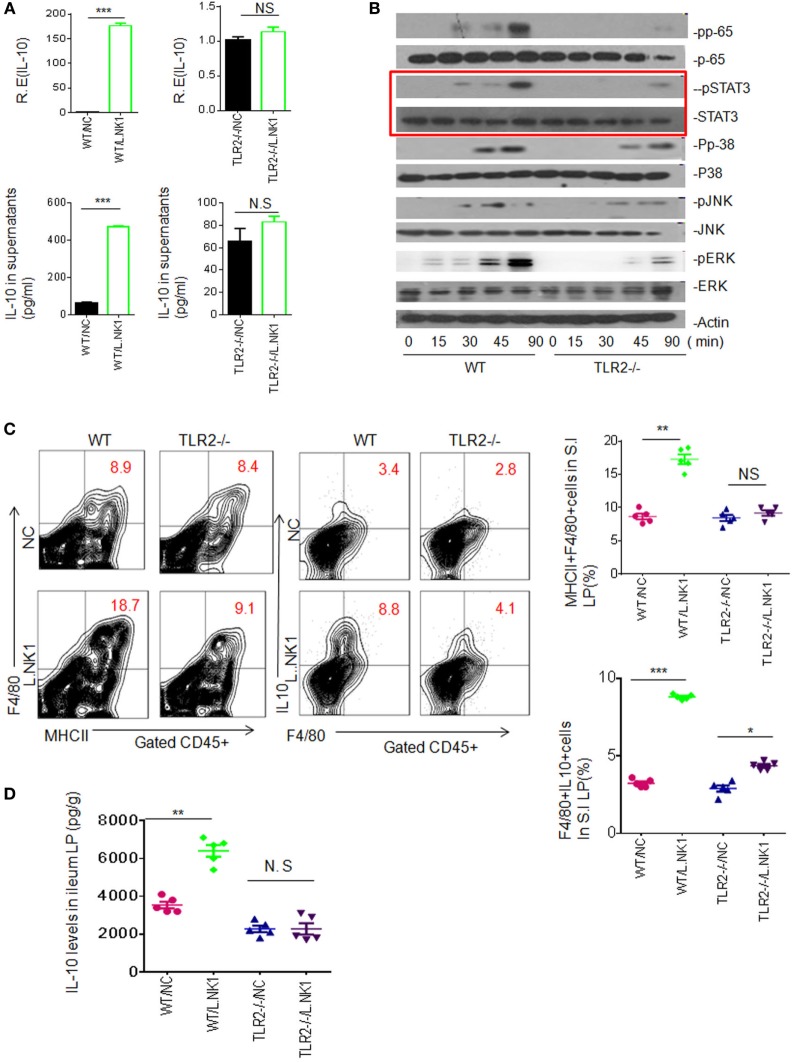
TLR2 is involved in *lactobacillus NK318.1*-mediated STAT3 activation. **(A)** qRT-PCR and ELISA (lower) of IL-10 in the wild-type (WT) and *TLR2^−/−^* macrophages in response to heated-dead *Lactobacillus 318.1*. Macrophages were isolated from the ileum LP. **(B)** Immunoblotting of pp65, pSTAT3, pp-38, pJNK, and pERK in the isolated macrophages from the ileum LP of WT and *TLR2^−/−^* mice after exposing to *Lactobacillus NK318.1*. **(C)** Flow cytometry of MHCII(+)F4/80(+) and F4/80(+)IL-10(+) macrophages in the small intestinal (S.I) LP of WT and *TLR2^−/−^* mice with (L.NK.1) or without (NC) *Lactobacillus NK318.1* gavage. The proportion of MHCII(+)F4/80(+) and F4/80(+)IL-10(+) macrophages in WT and *TLR2^−/−^* mice with (L.NK1) or without (NC) L.NK1 gavage were compared (right; *n* = 5). **(D)** Comparison of IL-10 in the ileum LP of WT and *TLR2^−/−^* mice with (L.NK.1) or without L.NK.1 (NC) gavage (*n* = 5). *p < 0.05, **p < 0.01, and ***p < 0.001 [*t*-test in **(A)**; one-way analysis of variance in **(C,D)**, mean ± SD]. NS, no significant; qRT-PCR, quantitative real-time PCR; pJNK, phosphor-JNK; pERK, phosphor-ERK; WT, wild-type; LP, lamina propria.

Since *Lactobacillus* belongs to Gram-positive bacteria, we also examined whether TLR2 was involved in the accumulation of *Lactobacillus NK318.1-*mediated macrophages. Heated-dead *Lactobacillus NK318.1* may induce IL-10 expression in the freshly isolated WT macrophages but not in TLR2^−/−^ macrophages (Figure 4A). These dead bacteria might not only activate multiple signal pathways such as NF-κB, JAK, and ERK but also activated STAT3, whereas these signal molecules in *TLR2^−/−^* macrophages were not or weakly activated (Figure 4B). *In vivo, Lactobacillus NK318.1* gavage caused the accumulation of macrophages in WT mice but not in *TLR2^−/−^* mice (Figure 4C). The levels of IL-10 in gut tissues of WT but not in *TLR2^−/−^* mice were significantly higher after *Lactobacillus* gavage (Figure 4D). All of these suggest that TLR2 is involved in *Lactobacillus*-induced macrophages in *huREG3*γ*^tgIEC^* mice and meanwhile also indicate that *Lactobacillus NK318.1* may directly activate STAT3 signal pathway.

**Figure 5:**
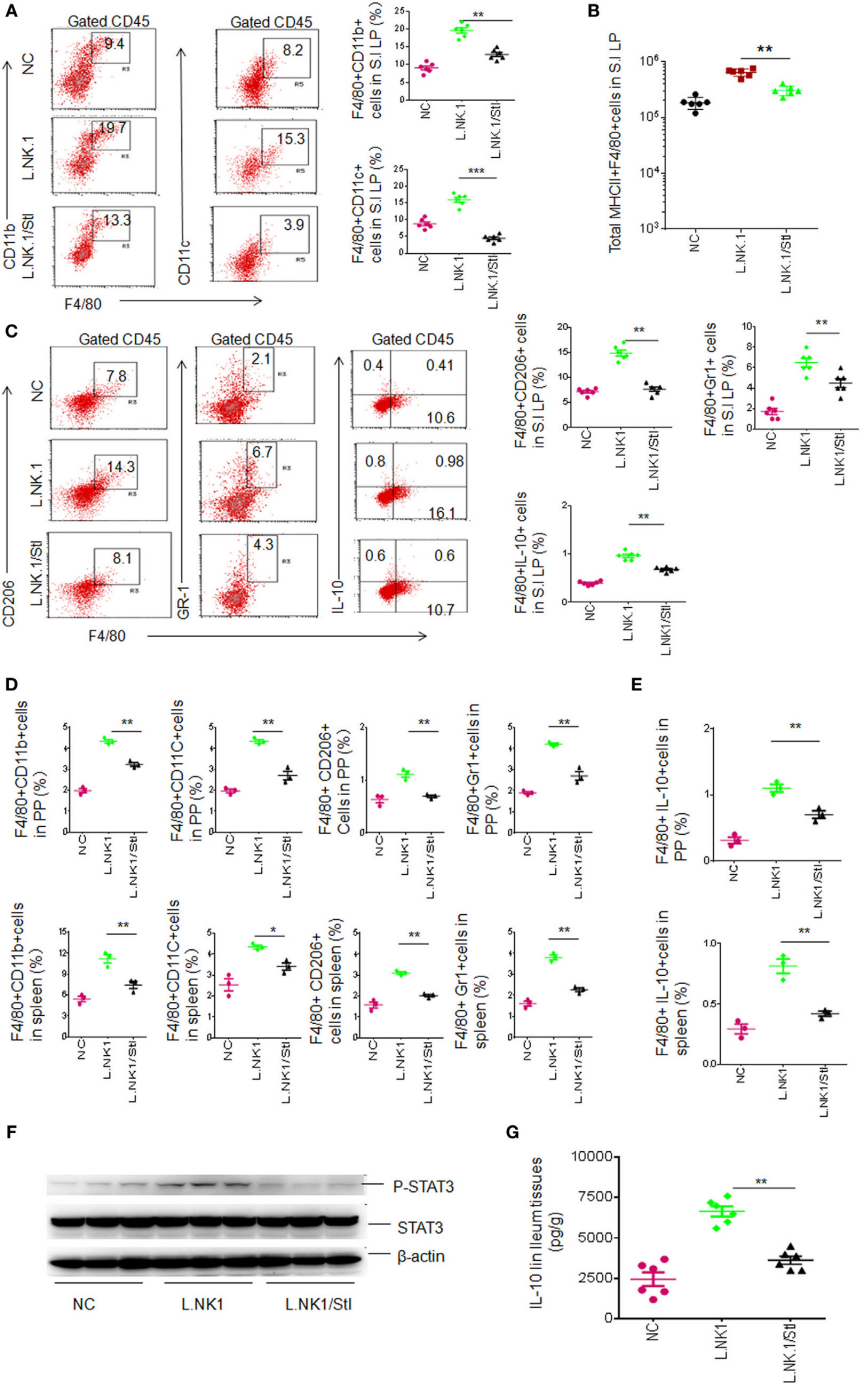
*Lactobacillus NK318.1-*mediated macrophages depend on STAT3. **(A)** Flow cytometry of F4/80(+)CD11b(+) and F4/80(+)CD11C(+) macrophages in the small intestinal (S.I) lamina propria (LP) of wild-type (WT) mice with (L.NK.1/StI) or without (L.NK.1) STAT3 inhibitor. F4/80(+)CD11b(+) and F4/80(+)CD11C(+) macrophage populations in the WT mice with (L.NK.1/StI) or without (L.NK.1) STAT3 were compared (right, *n* = 6). **(B)** Comparison of total F4/80(+)CD11b(+) macrophages in whole S.I LP of WT mice with (L.NK.1/StI) or without (L.NK.1) STAT3 inhibitor were compared (*n* = 6). **(C)** Flow cytometry of the macrophages in S.I LP of *huREG3*γ*^tgIEC^* and control littermate WT mice. F4/80(+)CD206(+), F4/80(+)Gr-1(+), and F4/80(+)IL-10(+) macrophages in the LP were compared (right, *n* = 6). **(D)** Comparison of F4/80(+)CD11b(+), F4/80(+)CD11C(+), F4/80(+)CD206(+), and F4/80(+)Gr-1(+) macrophages in the Peyer’ s patches (PP) and spleen of WT mice with (L.NK1/StI) or without (L.NK.1) STAT3 inhibitor were compared (*n* = 6). **(E)** Comparison of F4/80(+)IL-10(+) macrophages in the PP and spleen of WT mice with (L.NK1/StI) or without (L.NK.1) STAT3 inhibitor were compared (*n* = 6). **(F)** Immunoblotting of phosphor-STAT3 in the ileum tissues of WT mice with (L.NK1/StI) or without (L.NK.1) STAT3 inhibitor (number, different individuals). **(G)** IL-10 ELISA of ileum tissues in WT mice with (L.NK1/STAT3i) or without (L.NK1) STAT3 inhibitor (*n* = 6). NC, mice without *Lactobacillus* gavage and STAT3 inhibitor injection; L.NK.1, mice with *Lactobacillus* gavage but without injecting STAT3 inhibitor; L.NK.1/StI, mice with *Lactobacillus* gavage and STAT3 inhibitor injection. *p < 0.05, **p < 0.01, and ***p < 0.001 (one-way analysis of variance, mean ± SD). NS, no significance. Data for all panels are a representative from two to three experiments.

We next further determine STAT3 contribution in gut anti-inflammatory macrophages. STAT3 is regarded as one of the primary signaling molecules for macrophage polarization (30, 31). *In vitro* experiment revealed that STAT3 inhibitor impeded the *Lactobacillus*-mediated IL-10 expression (not shown), indicating that *Lactobacillus NK318.1* may affect the differentiation of gut macrophages. We next employed STAT3 inhibitor (JSI-124) to detect STAT3 effects on the macrophages *in vivo*. Injection of STAT3 inhibitor impeded *Lactobacillus NK318.1*-mediated increased macrophages. The proportion and total number of macrophages in the LP significantly decreased after injecting STAT3 inhibitor (Figures 5A,B). The proportion of the subsets such as MHCII(+)F4/80(+)CD11C(+)CD206(+), MHCII(+)F4/80(+)CD11C(+)Gr-1(+), and MHCII(+)F4/80(+)CD11C(+)IL-10(+) macrophages not only decreased in the LP but also in the PP and spleen after injecting STAT3 inhibitor (Figures 5C–E). Meanwhile, STAT3 activation was markedly reduced in the gut tissues of STAT3 inhibitor injected mice (Figure 5F). IL-10 levels in the ileum tissues of STAT3 inhibitor injected mice were also lower (Figure 5G). In addition, STAT3 inhibitor affects not only *Lactobacillus*-mediated macrophages but also the macrophages in WT and *huREG3*γ*^tgIEC^* mice. Taken together, we demonstrate that STAT3 plays a critical role in REG3γ-associated *Lactobacillus*-mediated accumulation of anti-inflammatory macrophages.

### Accumulated Anti-inflammatory Macrophages Impede HFD-Mediated Obesity

**Figure 6:**
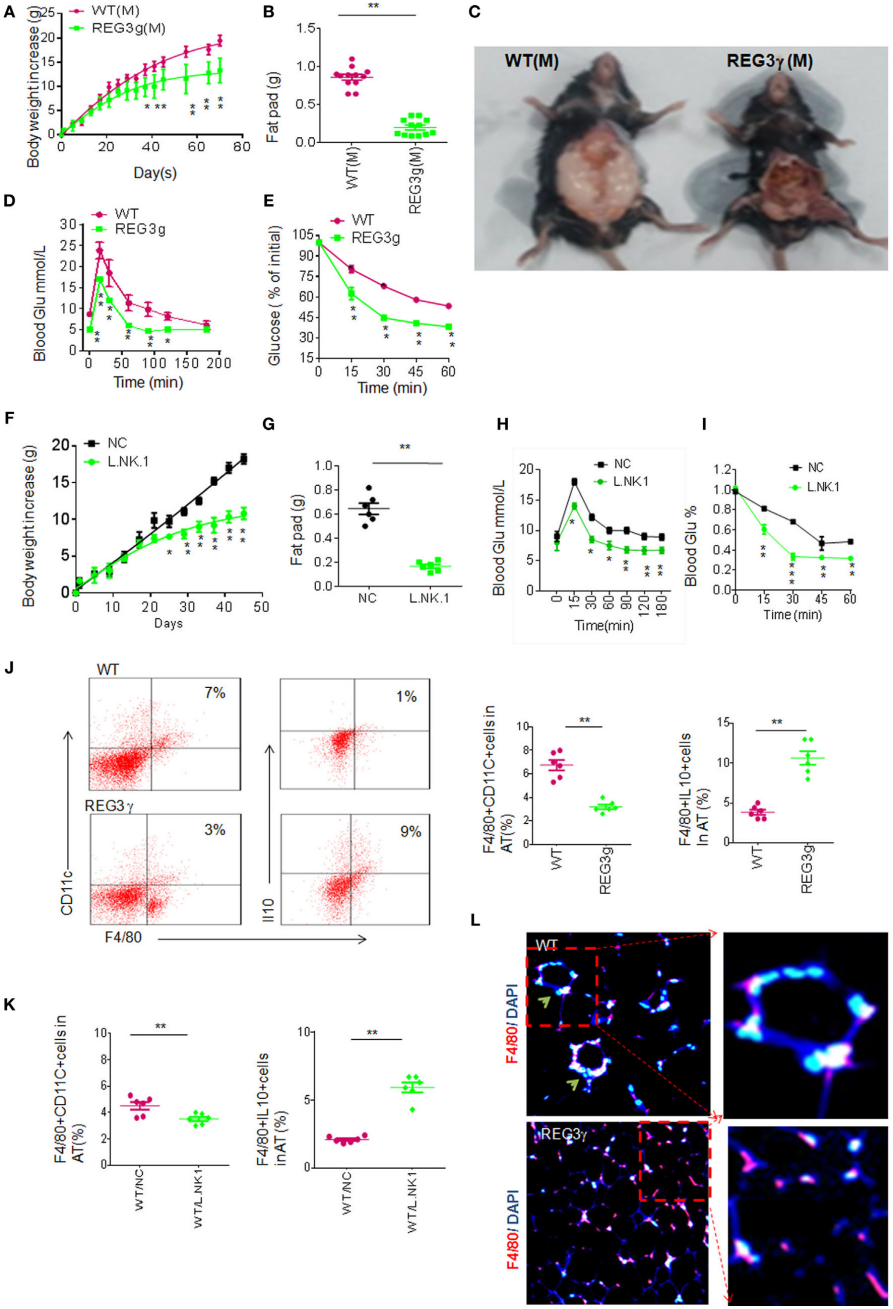
Accumulation of interleukin (IL)-10(+) macrophages in adipose tissues of *huREG3*γ*^tgIEC^* mice that are resistant to high-fat diet-mediated obesity. **(A)** Body weights of *huREG3*γ*^tgIEC^* and control littermate wild-type (WT) mice after high-fat diet. *huREG3*γ*^tgIEC^* (REG3g) and control littermate WT mice (*n* = 12, male) were observed and weighed at the indicated times after giving HFD. These mice are no differences at baseline before high-fat feeding. **(B,C)** Comparison of fat pad tissues of WT and *huREG3*γ*^tgIEC^*. The images in **(C)** were a representative from 12 mice. **(D,E)** Glucose **(D)** and insulin **(E)** tolerance in male *huREG3*γ*^tgIEC^* (REG3g) and control littermate WT mice after high-fat diet. WT and *huREG3*γ*^tgIEC^* (*n* = 6, male) mice after HFD for 3 months were analyzed for glucose and insulin tolerance. **(F,G)** Body **(F)** and fat pad **(G)** weights of mice with (L/NK.1) or without (NC) *Lactobacillus NK318.1* gavage (1 × 10^9^; once per week) during high-fat diet. **(H,I)** Glucose **(H)** and insulin **(I)** tolerance in mice with (L/NK.1) or without (NC). **(J)** Flow cytometry of macrophages in the adipose of *huREG3*γ*^tgIEC^* (REG3g) and control littermate WT mice. CD11C(+)F4/80(+) and F4/80(+)IL-10(+) cells in the adipose tissues were compared (right, *n* = 6). **(K)** Comparison of CD11C(+)F4/80(+) and F4/80(+)IL-10(+) cells in the adipose tissues of mice with (WT/L.NK.1) or without (WT/NC) *Lactobacillus* gavage (right, *n* = 6). **(L)** Immunostaining of F4/80(+) macrophages in the adipose of the *huREG3*γ*^tgIEC^* (REG3g) and control littermate WT mice. *p < 0.05, **p < 0.01, and ***p < 0.001: one-way analysis of variance in **(A,D,E,F,H,I)**; *t*-test in **(B,G,J,K)**. Data are a representative of three independent experiments.

The accumulated macrophages in *huREG3*γ*^tgIEC^* mice may cause reduced inflammation, which is often related to HFD-mediated obesity (2, 32, 33). We next employed HFD-mediated obesity model to detect the effects of accumulated macrophages in *huREG3*γ*^tgIEC^* mice on the obesity. We found that *huREG3*γ*^tgIEC^* mice had a remarkable resistance to HFD-mediated obesity and reduced sensitivity to insulin and glucoses compared to WT littermate mice (Figures 6A–E). *Lactobacillus NK318.1* gavages also impeded HFD-mediated obesity and reduced sensitivity to insulin and glucoses (Figures 6F–I). Interestingly, IL-10(+)F4/80(+) macrophages were much more in the adipose tissues of *huREG3*γ*^tgIEC^* mice than in the those of control WT littermate mice fed HFD (Figure 6J). Similar phenomenon was also found in *Lactobacillus* infused mice (Figure 6K). The macrophages in the adipose tissues of *huREG3*γ*^tgIEC^* mice or *Lactobacillus* infused mice also exhibited the anti-inflammatory phenotype (Figure 6L), which evenly distribute in adipose tissues (34). Because of critical role of anti-inflammatory macrophages in impeding obesity (34, 35), the resistance of *huREG3*γ*^tgIEC^* mice and *Lactobacillus* infused mice to HFD-mediated obesity may be related to accumulation of these anti-inflammatory macrophages.

**Figure 7:**
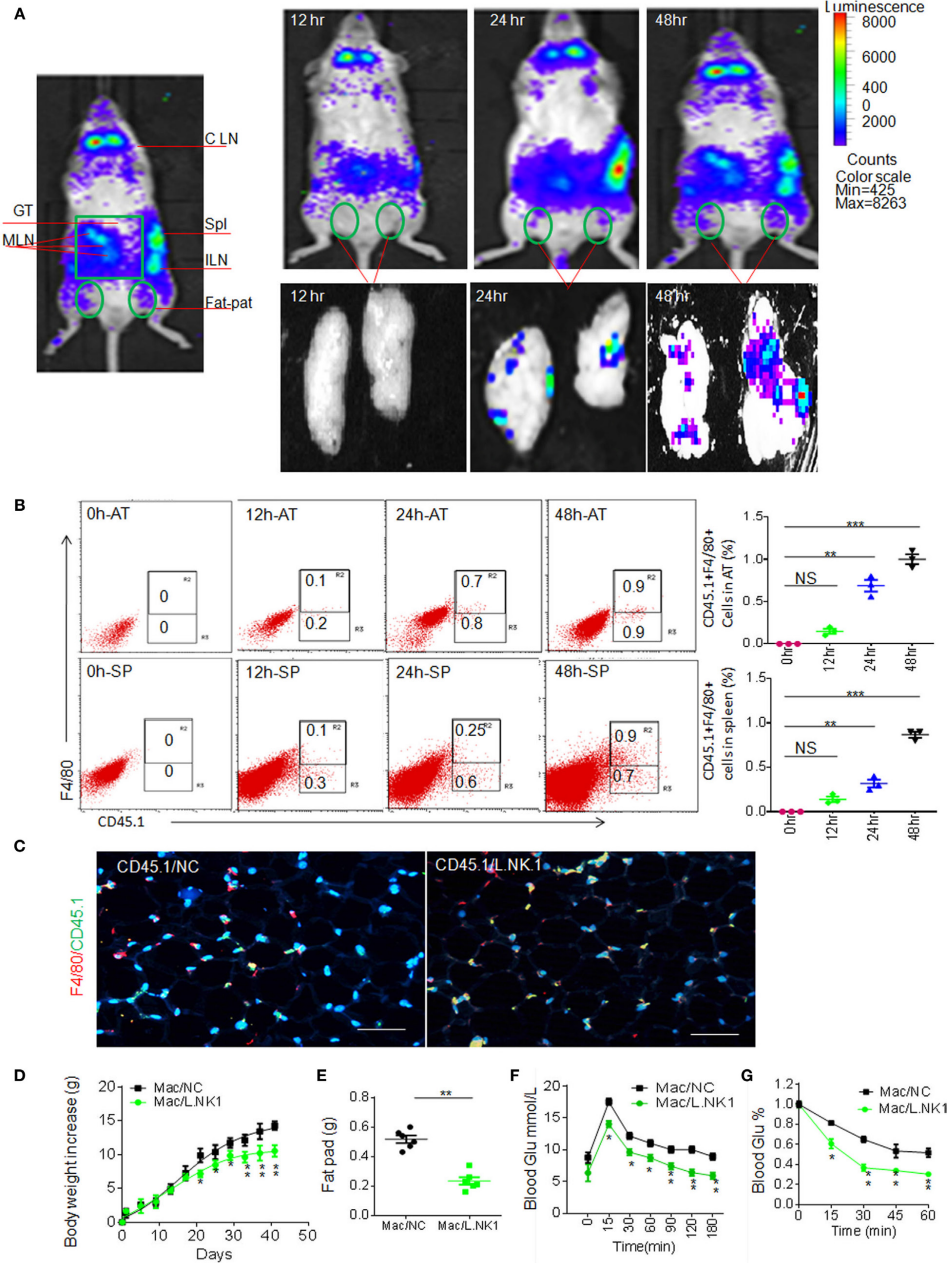
Accumulated macrophages in gut tissues are involved in resistance to high-fat diet-induced obesity. **(A)** Dynamics of macrophages from the ileum tissues of HFV/N (Lu-actin) mice with *Lactobacillus NK318.1* gavage. The macrophages isolated from the gut tissues of HFV/N (Lu-actin) mice with *Lactobacillus NK318.1* gavage were injected into WT mice, and then macrophages in the adipose tissues of WT mice were analyzed at the indicated time. **(B)** Flow cytometry of CD45.1(+)F4/80(+) macrophages in the adipose tissues and spleen at the different times after injecting gut macrophages of CD45.1(+) mice with *Lactobacillus NK318.1* gavage. AT, adipose tissues; SP, spleen. **(C)** Immunostaining of macrophages in the adipose tissues after injecting gut lamina propria macrophages of CD45.1(+) mice with (CD45.1/L.NK.1) or without (CD45.1/NC) *Lactobacillus NK318.1* gavage. **(D–G)** Body weights **(D)**, fat pad **(E)**, glucose **(F)**, and insulin tolerance **(G)** in mice injecting freshly isolated macrophages from the ileum tissues of mice with (Mac/L.NK.1) or without (Mac/NC) *Lactobacillus* NK318.1 gavage. *p < 0.05, **p < 0.01, and ***p < 0.001 [one-way analysis of variance in **(B,D,F,G)**; *t*-test in **(E)**, mean ± SD]. NS, no significance; data for all panels are a representative from two to three experiments.

Next question is whether the accumulation of anti-inflammatory macrophages in adipose tissues is related to REG3γ-associated *Lactobacillus*. Previous data showed that gut immune cells possess broad trafficking abilities to lymphoid organs such as the inguinal lymph nodes and spleen (29). We next tested whether *Lactobacillus*-mediated macrophages might migrate into adipose tissues. To demonstrate this, we employed the *β-Actin-luc* LPTA^®^ animal model to examine the movement of *Lactobacillus NK318.1*-mediated macrophages. The isolated macrophages from the LP tissues of *β-Actin-luc* LPTA mice with *Lactobacillus NK318.1* orally gavage were transplanted into syngeneic recipients. Bioluminescent foci were detected as early as 12 h after transplantation and the most frequently observed locations were at anatomic sites corresponding to the locations of the spleen and gut tissues (Figure 7A). However, these gut immune tissue-derived macrophages may migrate into pat-pad adipose tissues (Figure 7A). While isolated CD45.1(+) macrophages from gut LP tissues were injected into congenic CD45.2(+) WT mice, CD45.1(+) macrophages could move into the pat-pad adipose tissues of mice (Figures 7B,C). Infusion of isolated macrophages from *Lactobacillus*-infused mice could impede the HFD-mediated obesity and reduced sensitivity to insulin and glucoses (Figures 7D–G). Finally, we also investigated the proportion of *Lactobacillus NK318.1* in obese and thin individuals. We found that the copies of *Lactobacillus NK318.1* in thin individuals were remarkably increased, whereas the copies of *Lactobacillus NK318.1* in obese individuals were less compared to normal individuals (Figure S6 in Supplementary Material). Taken together, our data suggest that the *Lactobacillus NK318.1*-associated macrophages are involved in the occurrence and development of obesity.

## Discussion

Intestinal-resident macrophages play a central role in the maintenance of homeostasis in the gastrointestinal tract (8, 36). Although there are the most abundant sources of resident tissue macrophages in the LP of gut tissues (7), the characteristics of these cells are not completely understood. Previous studies indicated that gut-resident macrophages do not fit readily into this “M1–M2 paradigm,” having some of hallmarks of both M1 and M2 macrophages (37). Recent studies also indicate that tissue-resident macrophages are characterized as MHCII(+)CD11C(+)CD103(−)CD11b(+)CX3CRl(+)F4/80(+)CD64(+) cells (6). We here also found that these macrophages include multiple subpopulations such as F4/80(+)CD206(+), F4/80(+)CD206(−), F4/80(+)Gr-1(+), F4/80(+)Gr-1(−), F4/80(+)IL-10(+), and F4/80(+)IL-10(−) in small intestinal LP tissue of mice.

The interactions with different components of microbiota are crucial to the establishment and development of gut immune cell populations (5, 13–16). The intestinal mucosa contains one of the largest populations of macrophages. However, it is unclear how gut microbes regulate these anti-inflammatory macrophages in gut tissues. The maintenance of intestinal macrophages requires constant *de novo* migration of Ly6C(hi)CCR2(+) blood monocytes to intestinal tissues for replenishment (38). However, the presence of gut microbiota plays a crucial role in this replenishment of macrophages (9, 39). We demonstrate that *Lactobacillus NK318.1* may induce the differentiation of gut macrophages and expand anti-inflammatory macrophage pools. *In vitro* experiment found that *Lactobacillus* teichoic acids may reverse IL-12 into predominant IL-10 production *via* TLR2-dependent ERK activation in macrophages (40). The colonization of GF mice with a mixture of three *Lactobacillus* strains enhances the integrity of gut mucosa and ameliorates allergic sensitization (41). Thus, some gut *Lactobacillus* strains may be critical in maintaining homeostasis of gut macrophages. However, what is the molecular nature of the *Lactobacillus*-provided ligand that binds to TLR2, driving differentiation into anti-inflammatory macrophages, need to be clear.

We found that STAT3 is a critical factor in the differentiation of *Lactobacillus NK318.1*-mediated anti-inflammatory macrophages. STAT3 is important for cell proliferation, survival, and motility (42) and is regarded as one of the primary signaling molecules for macrophage polarization (43). It may be activated by phosphorylation at *C*-terminal Tyr 705 by JAKs, Ser 727 by protein kinase C, mitogen-activated protein kinases, or CDK5. Macrophages from STAT3 knockout mice release high levels of pro-inflammatory cytokines (44). STAT3 deficiency may not only affect macrophages but also affect neutrophils (45). The ablation of STAT3 expression through the use of conditional knockout mice or selective STAT3 inhibitor significantly reduced the expansion of MDSCs and increased T cell responses in tumor-bearing mice (46).

Traditional concept is that blood monocytes could migrate into adipose tissues, where they are differentiated into M1 or M2 to regulate the adipose inflammation to affect development of obesity (11). However, the origin of adipose tissue macrophages in lean (M2 macrophages) and obese adipose tissue (M1 macrophages) is still a matter of debate. We here found that gut anti-inflammatory macrophages could move into adipose tissues. Thus, our data suggest that anti-inflammatory macrophages in adipose tissues of thin individuals may come from the gut tissues. Kupffer cells, one kind of macrophages in the liver, also argue that the macrophages originate from local intrahepatic progenitors (47). Bowel immune cells, including CD4(+) T cells, Treg, and Th17, have been shown to possess broad trafficking abilities to lymphoid organs at distant sites, such as the inguinal lymph nodes and spleen (29). Thus, all of these may imply a new mechanism how gut microbiota control the development of obesity. However, whether the migration of anti-inflammatory macrophages into adipose tissue occurs naturally needs to be further investigated.

In conclusion, we show that gut anti-inflammatory macrophage populations are consisted of multiple cell subpopulations. We demonstrate that REG3γ-associated *Lactobacillus NK318.1* in *huREG3*γ*^tgIEC^* mice may enlarge macrophage pools not only in gut tissues but also in spleen and adipose tissues. The anti-inflammatory macrophages induced by gut *Lactobacillus NK318.1* in gut LP may migrate into adipose tissues. Our study not only helps to elucidate the role of REG3γ-associated *Lactobacillus* in expanding gut anti-inflammatory macrophage pools but also provides a new insight into the understanding of anti-inflammatory macrophages in the adipose tissues of thin individuals, which is critical for developing new strategies against obesity.

## Ethics Statement

All animal studies were conducted according to the Institutional Animal Care and Use Committee of the Model Animal Research Center. Animal experiments were approved by the Institute’s Animal Ethics Committee of Nankai University.

## Author Contributions

RY designed the research and wrote the paper; YH, HQ, ZZ, EW, Huan.Y, XS, and Hui.Y conducted *in vivo* and *in vitro* experiments and immunoassay, participated in the study design, and performed the statistical analysis; YL, ZT, YG, WS, and TW performed the *in vitro* assay and YC, JZ, and YZ offered assistances for the animal experiments. All authors read and approved the final manuscript.

## Conflict of Interest Statement

The authors declare that the research was conducted in the absence of any commercial or financial relationships that could be construed as a potential conflict of interest.
