# Supplementary material for: Gut REG3γ-Associated Lactobacillus Induces Anti-inflammatory Macrophages to Maintain Adipose Tissue Homeostasis
Source: Front Immunol. 2017 Sep 4;8:1063. doi: 10.3389/fimmu.2017.01063 (PMC5591335; doi:10.3389/fimmu.2017.01063)
Supplement: Supplementary file 1 [file Presentation_1.PDF]

Supplemental Information for

Gut REG3γ associated *Lactobacillus* Induces Anti-inflammatory Macrophages to Maintain Adipose Tissue Homeostasis

Yugang Huang[1*], HouBao Qi[1*], Zhiqian Zhang[1*], Enlin Wang[1*], Huan Yun[1], Hui Yan[1], Xiaomin Su[1], Yingquan Liu[1], Zenzen Tang[1], Yunhuan Gao[1], Wencong Shang[1], Jiang Zhou[1], Tianze Wang[1], Yongzhe Che[1], Yuan Zhang[1] and Rongcun Yang[1, 2, 3]

[1]State Key Laboratory of Medicinal Chemical Biology; [2]Key Laboratory of Bioactive Materials Ministry of Education; [3]Department of Immunology, Nankai University School of Medicine; Nankai University, Tianjin, China.

Supplementary Table S1.

Table S1. Primers Used in This Manuscript.

(A) Primers used in the detection of genes.

Oligo name	Sequence (5' to 3')	Description
Murine GAPDH-s<br>Murine GAPDH-as	TCAACGGCACAGTCAAGG<br>TACTCAGCACCGGCCTCA	115 bp
Murine β-actin-s<br>Murine β-actin-as	ATCATTGCTCCTCCTGAGCG<br>GCTGATCCACATCTGCTGGAA	90 bp
Murine Reg3γ-s<br>Murine Reg3γ-as	TTCCTGTCCTCCATGATCAAA<br>CATCCACCTCTGTTGGGTTC	101 bp
Homo Reg3γ-s<br>Homo Reg3γ-as	TGAAGAAACCCAGAAGGAACTGC<br>GAGGACACGAAGGATCCCTCA	192bp
Murine TNFα-s<br>Murine TNFα-as	GGTCTGGGCCATAGAACTGA<br>CAGCCTCTTCTCATTCCTGC	133 bp
Murine IL-6-s<br>Murine IL-6-as	TCTGAAGGACTCTGGCTTTG<br>GATGGATGCTACCAAACTGGA	142 bp
Murine IL-1β-s<br>Murine IL-1β-as	GTGTCTTTCCCGTGGACCTT<br>AATGGGAACGTCACACACCA	129 bp
Murine IL-17a-s<br>Murine IL-17a-as	ACTACCTCAACCGTTCCACG<br>ATGTGGTGGTCCAGCTTTCC	136 bp
Murine IL-12b-s<br>Murine IL-12b-as	TGGTTTGCCATCGTTTTGCTG<br>ACAGGTGAGGTTCACTGTTTCT	123 bp
Murine Il10-s<br>Murine Il10-as	CCAAGCCTTATCGGAAATGA<br>TTTTCACAGGGGAGAAATCG	163 bp
Murine IL-1m-s<br>Murine IL-1m-as	TTGGATCATGGCAGGTGCTT<br>GTAAGGGAGTCACTTGGGGC	222

(B) Primers used in the identification of gut microbiota.

Oligo name	Sequence (5’-3’)	Description
Bacteroides Phylum	F: GAGAGGAAGGTCCCCCAC<br>R: CGCTACTTGGCTGGTTCAG	rRNA-16S
Firmicutes Phylum	F:GCTGCTAATACCGCATGATATGTC<br>R:CAGACGCGAGTCCATCTCAGA	rRNA-16S
Lactobacillus	F: AGCAGTAGGGAATCTTCCA<br>R: CACCGCTACACATGGAG	rRNA-16S
Eubacteria	F: AGCAGTAGGGAATCTTCCA<br>R: CACCGCTACACATGGAG	rRNA-16S
L. NK318.1	F: CATCCAGTGCAAACCTAAGAG<br>R: GATCCGCTTGCCTTCGCA	rRNA-16S
L.NK318.2	F: AGCTAGTTGGTGAGGTAAAG<br>R: TAGGATTGTCAGAAGATGTC	rRNA-16S
L. reuteri	F: ACCGAGAACACCGCGTTATTT<br>R:CATAACTTAACCTAAACAATCAAAGATTGTC<br>T	rRNA-16S
L. gasseri	F:AGCGAGCTTGCCTAGATGAATTTG<br>R:TCTTTTAAACTCTAGACATGCGTC	rRNA-16S
L. animals	F:AGCTAGTTGGTGAGGTAAAG<br>R:TAGGATTGTCAGAAGATGTC	rRNA-16S
L. rhamnosus	F: CTAGCGGGTGCGACTTTGTT<br>R: GCGATGCGAATTTCTATTATT	rRNA-16S
L. plantarum	F:CTCTGGTATTGATTGGTGCTTGCAT<br>R: GTTCGCCACTCACTCAAATGTAAA	rRNA-16S
Lactobacillus Probes	FAM-O-ACATGGAGTTCCACT	

Supplementary Figures

Figure S1. Related to Figure 1 to Figure 7

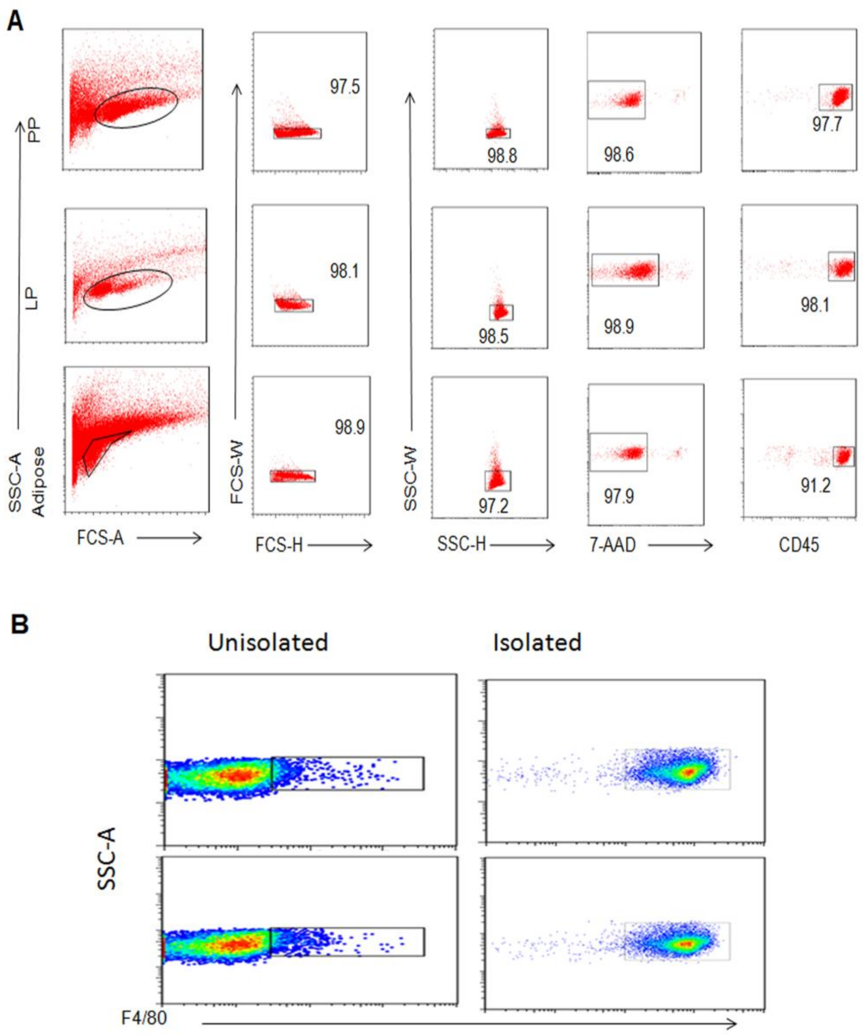

Figure S1. Representative FACS Gating Schem for Immune Cell Analyses in LP, PP and Adipose Tissues (A), and Flow Cytometry of Isolated Macrophages from Small Intestinal LP (B). The macrophages were isolated using anti-F4/80 magnetic beads with two cycles.

Notably, macrophages in the LP, PP, MLN and spleen were not stained by FITC-, PE-,

APC- APC/cy7-, PerCP/Cy5.5- or PE/cy7-conjugated isotypic antibody control.

Figure S2. Related to Figure 1.

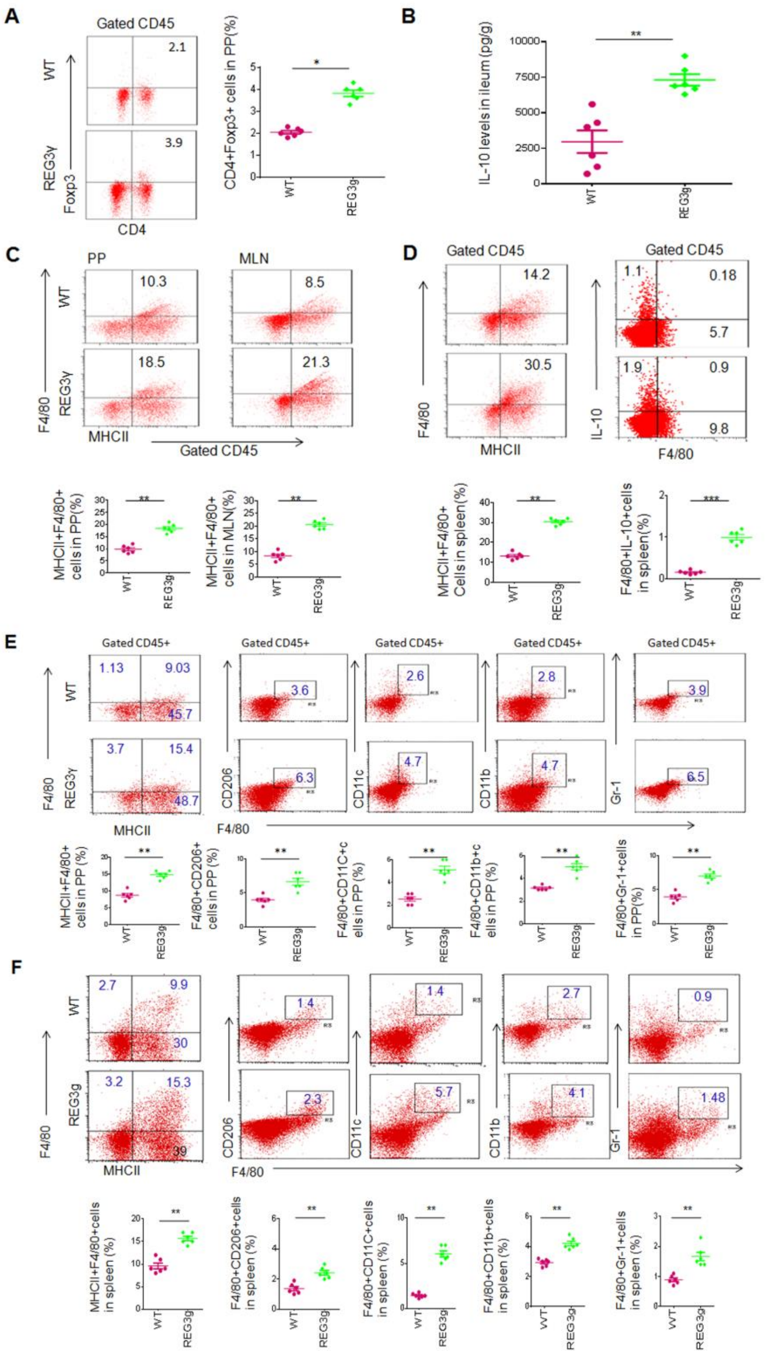

Figure S2. Macrophages in Payer's Patch and Spleen of *HuREG3γ* ^{tgIEC} Mice.

(A) Flow cytometry of CD4(+)Foxp3 (+) cells in small intestinal LP of huREG3γ ^{tgIEC} and control littermate WT mice. Proportion of CD4(+)Foxp3(+) cells in WT and huREG3γ ^{tgIEC} mice (REG3γ) were compared (n=6, right). (B) ELISA of IL-10 in

ileum tissues of huREG3γ ^{tgIEC} and control littermate WT mice. (C) Flow cytometry of MHCII(+)F4/80(+) macrophages in Payer patch (PP) and mesenteric lymphoid node (MLN) of huREG3γ ^{tgIEC} (REG3g) and control littermate WT mice. MHCII(+)F4/80(+) macrophages in WT and huREG3γ ^{tgIEC} mice were compared (n=6, lower). (D) Flow cytometry of MHCII(+)F4/80(+) and F4/80(+)IL-10(+) macrophages in the spleen of huREG3γ ^{tgIEC} and control littermate WT mice. MHCII(+)F4/80(+)and F4/80(+)IL-10(+) macrophages in WT and huREG3γ ^{tgIEC} mice (REG3g) were compared (n=6, lower;). (E and F) Flow cytometry of the macrophages in Payer's patch (E) and spleen (F) of *huREG3γ*^{tgIEC} (REG3g) and control littermate WT mice. Gated $CD45^{+}$ cells were analyzed by staining using surface markers MHCII, F4/80, CD11b, CD11C, Gr-1 and CD206 after double and dead cells were gated out according to supplementary Figure S2A. MHCII(+)F4/80(+), F4/80(+)CD206(+), F4/80(+)CD11C(+), F4/80(+)CD11b(+), F4/80(+)Gr-1 (+) cells in payer's patch and spleen were compared (n=6) .

*P<0.05, **P<0.01 and *** P<0.001 (t-test, mean ± SD). N.S, no significance; Data for all panels is a representative from two to three experiments.

Figure S3: Related to Figure 1.

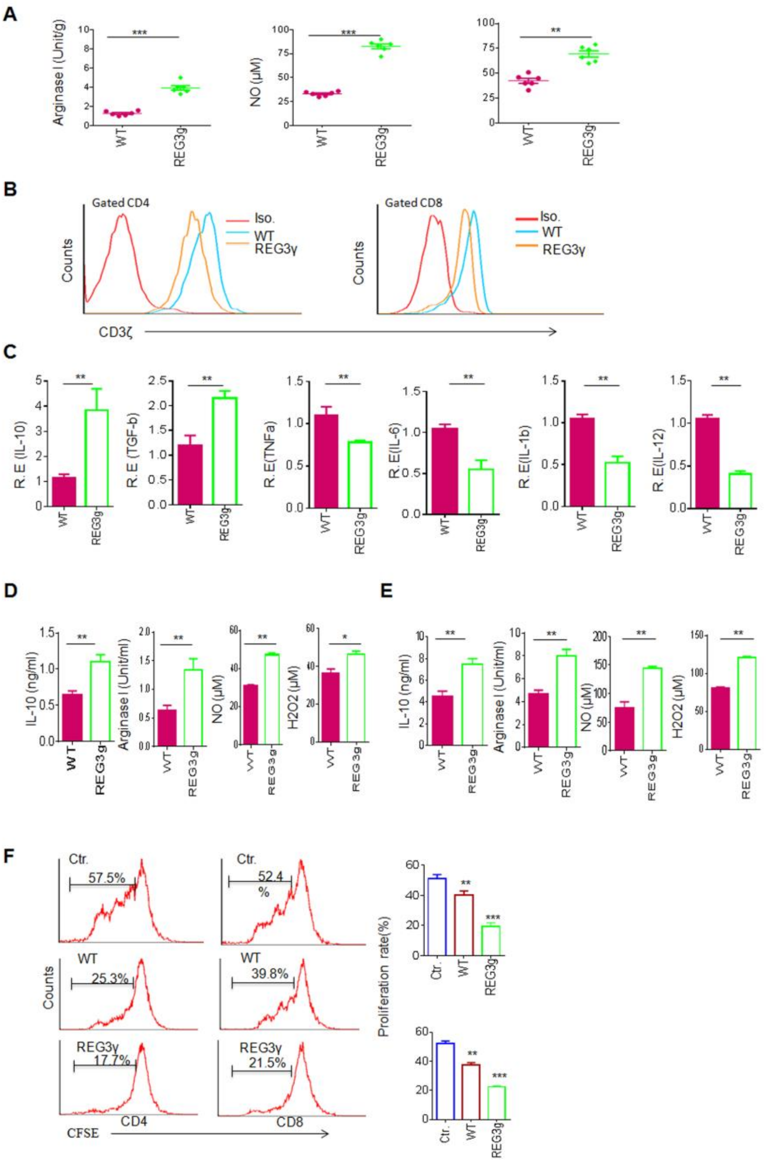

Figure S3. REG3γ Promotes Homeostasis of Small Intestines.

(A) Assays of arginase-1, H2O2 and NO in the ileum tissue per gram of *huREG3γ* ^{tgIEC} (REG3g) or control littermate WT mice (n=6). Gut tissues was milled in liquid nitrogen and then lysed with non-denatured tissue lysis solution for assays. (B) Flow cytometry analyses of CD4 and CD8 zeta chain in the ileum tissues of *huREG3γ* ^{tgIEC}

(REG3g) and control littermate WT mice. One representative from 6 samples. (C) qRT-PCR of IL-10, TGFβ, TNFα, IL-6, IL-1β and IL-12 in the ileum tissues of *huREG3γ*^{tgIEC} and control littermate WT (n=6). (D and E) Analyses of IL-10, arginase-1, NO and H2O2 in the supernatants of isolated macrophages with (D) or (E) without stimulation of heated dead *Lactobacillus NK318.1* (Macrophages: *Lactobacilli*: 1:20). Macrophages were isolated from WT and *huREG3γ*^{tgIEC} mice (REG3γ) using magnetic beads. (F) Proliferation rate (%) of CD4 and CD8 in the presence of macrophages isolated from WT or *huREG3γ*^{tgIEC} mice (REG3γ). Proliferation rate (%) of CD4 and CD8 in the presence of macrophages isolated from WT or *huREG3γ*^{tgIEC} mice (REG3g) were compared (n=6). Ctr.. without macrophages *P<0.05, **P<0.01 and *** P<0.001 (*t-test* in A, C, D and E, mean ± SD; one-way analysis of variance in F). N.S, no significance; Data for all panels is a representative from two to three experiments.

Figure S4: Related to Figure 2

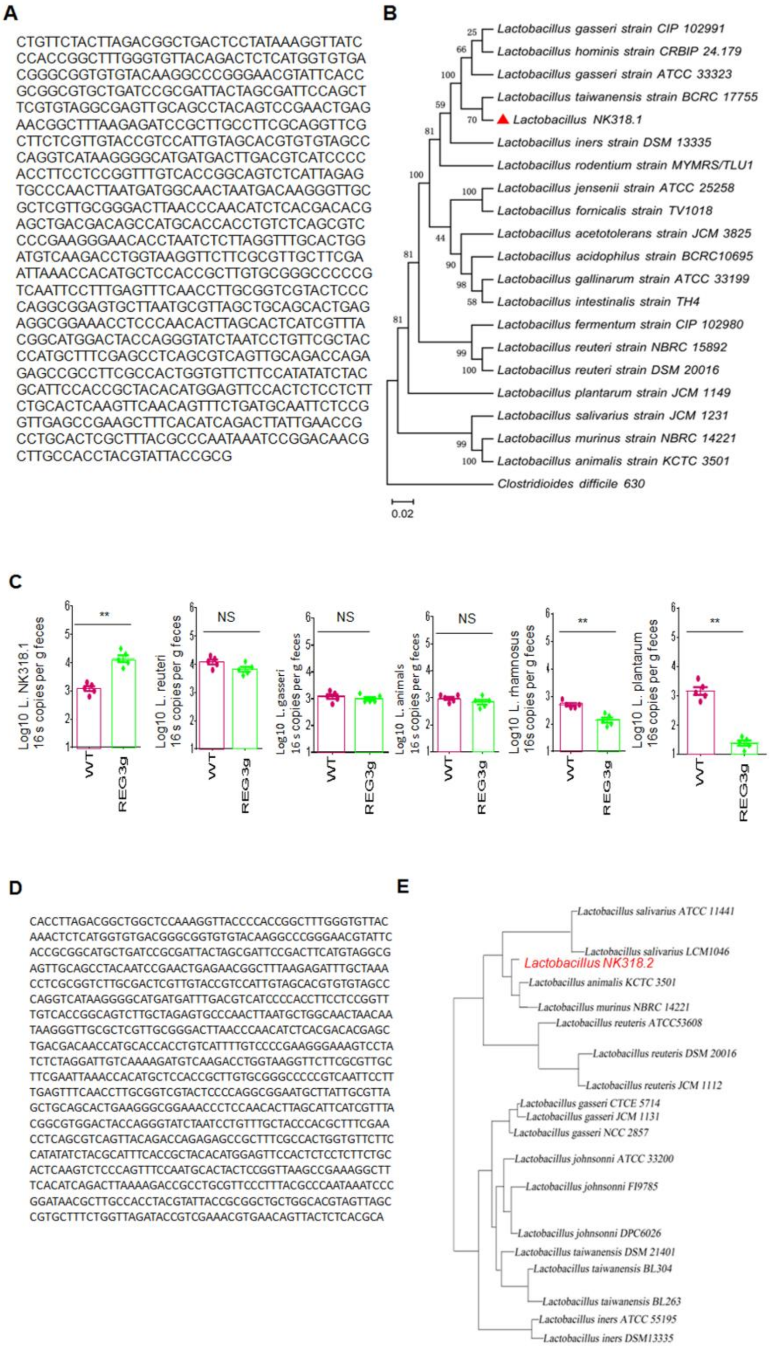

Figure S4. Characteristics of *Lactobacillus NK.1* and *Lactobacillus NK.2* 16s rRNA. (A) Sequence of *Lactobacillus NK318.1*. The 16s rRNAs from *Lactobacillus NK318.1* were extracted and sequenced by primers (Forward, 5'-AGCAGTAGGGAATCTTCCA; Reverse, 5'-CACCGCTACACATGGAG). *Lactobacilli* were isolated using Rogosa SL selective medium. Colonies were identified and purified for 16s rRNA sequence analyses for speciation of colonial genotype. The lactobacilli were further cultured in MRS media and also grown on

MRS agar containing 10% sucrose. Anaerobic conditions were generated with sachets of AnaeroPack-Anaero in an air-tight jar. (**B**) Phylogenetic tree showing relationships among 16S rRNA gene sequences of species in the *Lactobacillus NK318.1* group and species representing different lineages within the genus *Lactobacillus*. Numbers at nodes represent bootstrap percentages obtained by repeating analysis 1000 times. Scale bar = 0.02 estimated substitutions per site. Clostridioides, outgroup bacteria. (**C**)16S rRNA analyses of different *Lactobacillus* strains in the feces of $huREG3\gamma^{tgIEC}$ (REG3γ) and control littermate WT mice (n=5, male) fed normal chow. 16S rRNA copies of *Lactobaclillus NK318.1, Lactobacillus reuteri, Lactobacillus gasseri, Lactobacillus animals, Lactobacillus rhamnosus* and *Latobacillus plantarum* were analyzed using q-PCR. (**D**) Sequence of *Lactobacillus NK318.2*. The 16s rRNAs from *Lactobacillus* were extracted and sequenced by primers (F:
5’-AGAGTTTGATCATGGCTCAG-3’
R: 5’-TAGGGTTACCTTGTTACGACTT-3’). This strain *Lactobacillus* was named as *Lactobacillus NK318.2.* (**E**) Homology of isolated *Lactobacillus NK318.2* with other bacteria. The gut *Lactobacillus* were selected and cultured in *Lactobacillus* selected medium (Barebio, China). Phylogenetic tree showing relationships among 16S rRNA gene sequences of species in the *Lactobacillus animal* isolates, including *Lactobacillus* and species representing different lineages within the genus *Lactobacillus*.
*P<0.05, **P<0.01 and *** P<0.001 (t-test, mean ± SD). N.S, no significance; Data for all panels is a representative from two to three experiments.

Figure S5. Related to Figure 2.

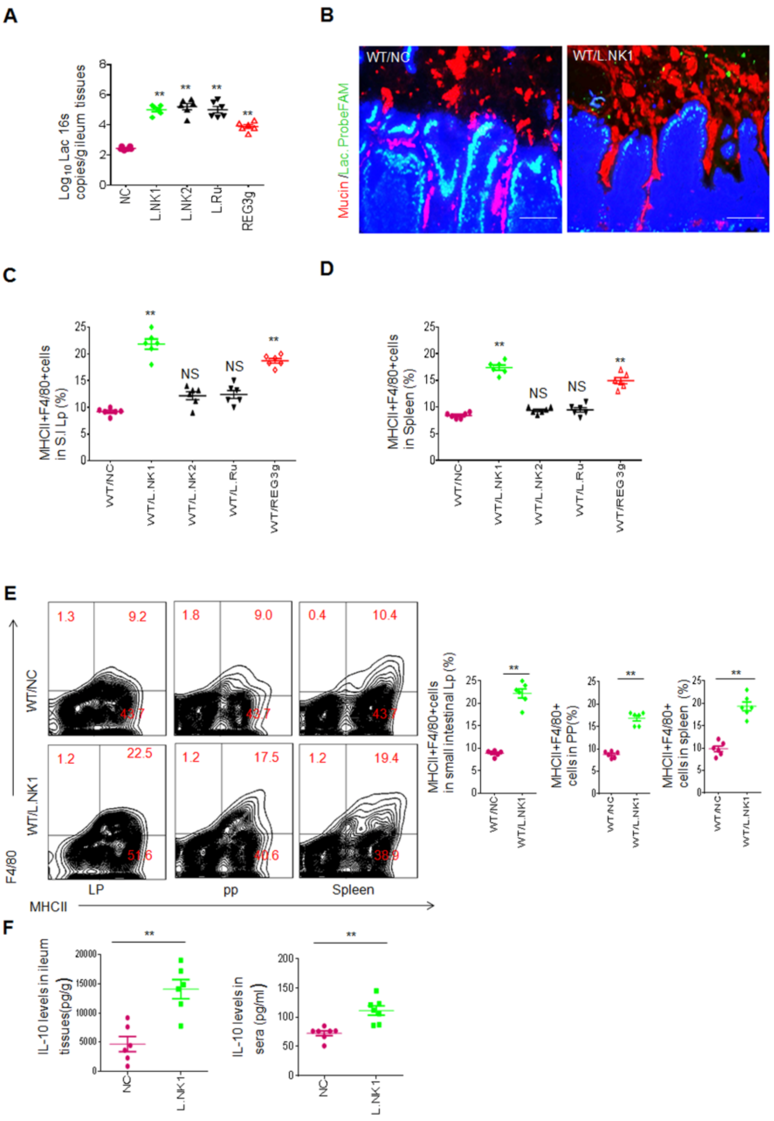

Figure S5. REG3γ-Associated *Lactobacillus NK318.1* Induces MHCII(+)F4/80(+)CD11C(+) Macrophages in Pan-Antibiotics Treated WT Mice. (A) Q-PCR of *Lactobacillus* in the ileum tissues of mice with *Lactobacillus NK318.1* (WT/L.NK1), *Lactobacillus NK318.2*(L.NK.2), *Lactobacillus reuteri* (L.Ru) and *huREG3γ*^{tgIEC} feces (REG3 γ) or without (WT/NC) orally gavage with *Lactobacillus NK318.1*. (B) Fluorescence in situ hybridization and immunostaining of ileum fragments in mice with (WT/L.NK.1) or without (WT/NC) orally gavage with

Lactobacillus NK318.1. Ileum fragments of mice were prepared for fluorescence in situ hybridization by fixation in Carnoy's fixative. Tissue sections were stained by anti-mucin 2 antibody (red) and hybridized to a probe (Lac663：FAM-O-ACATGGAGTTCCACT) that recognizes the Lactobacillus 16S rRNA (green), a non-specific scrambled probe and counterstained with DAPI to visualize nuclei (Blue). Scale bars=40 μm. (C and D) Comparison of the macrophages in the LP (C) and spleen (D) of mice orally infused with different kinds of *Lactobacillus*. The cells from the spleen of the mice after orally gavage with different *Lactobacillus* strains (1×10^9, once/week for three times) were stained using the indicated antibodies and analyzed using flow cytometry. (E) Flow cytometry of MHCII(+)F4/80(+) macrophages in the LP, PP and spleen mice with (WT/L. NK.1) or without (WT/NC) orally garage with *Lactobacillus NK318.1*. MHCII(+) F4/80(+) cell populations in mice with (L.NK.1) or without (NC) *Lactobacillus NK318.1* (n=6) were compared on the right. (F) Assays of IL-10 in the ileum tissues and sera of WT mice with (L. NK1) or without (NC) orally gavage with *Lactobacillus NK318.1*.
*P<0.05, **P<0.01 and *** P<0.001 (t-test, mean ± SD). Data are a representative of three independent experiments.

Figure S6.

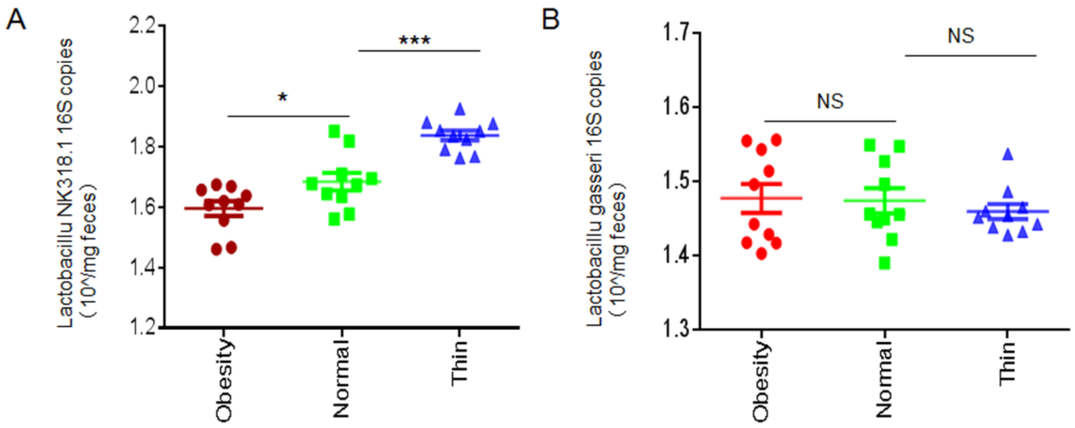

Figure S6. *Lactobacillus NK318.1* Is Related to Obesity in Human. Q -PCR of *Lactobacillus NK318.1* (A) and *Lactobacillus gasseri* (B) in normal (BMI (Body Mass Index): 18.5-23.9), obesity (BMI, 28-32) and thin (BMI less than 18.5) individuals (n=15, 23-24 years old male individuals without using antibiotics past one month).
